# Skilful probabilistic medium-range precipitation and temperature forecasts over Vietnam for the development of a future dengue early warning system

**DOI:** 10.1002/met.2222

**Published:** 2024-08-07

**Authors:** Lucy Main, Sarah Sparrow, Antje Weisheimer, Matthew Wright

**Affiliations:** 1 Department of Physics, University of Oxford, Oxford, UK; 2 Department of Engineering Science, University of Oxford, Oxford, UK; 3 European Centre for Medium-Range Weather Forecasts (ECMWF), Reading, UK

**Keywords:** CRPSS, dengue fever, ensemble forecasting, precipitation, rank histograms, temperature, verification, Vietnam

## Abstract

Dengue fever is a source of substantial health burden in Vietnam. Given the well-established influence of temperature and precipitation on vector biology and disease transmission, predictions of meteorological variables, such as those issued by ECMWF as a world-leading provider of global ensemble forecasts, are likely to be valuable model inputs to a future dengue early warning system. In the absence of established verification at municipal and regional scales, this study assesses the skill of rainy season (May–October) ensemble precipitation and 2-m temperature retrospective forecasts over North and South Vietnam initialized for dates during the period 2001–2020, evaluated against the ERA5 reanalysis for the same period. Forecasts are found to be significantly skilful compared with both climatology and persistence for lead times up to 10 days, including for cumulative precipitation values considered against independent rain gauge data. Rank histograms demonstrate that ensembles generally avoid excessive bias and consistently positive CRPSS values indicate substantial skill for temperature and cumulative precipitation forecasts for all spatial scales considered, despite differences in rainy season characteristics between North and South Vietnam. This forecast reliability demonstrates that meteorological input data based on ECMWF ensemble forecasts would add appreciably more value to the development of a future dengue early warning system compared to reference forecasts like climatology or persistence. These results raise hope for further exploration of predictive skill for relevant meteorological variables, particularly focused on their downscaling to produce district-level epidemio-logical forecasts for urban areas where dengue is most prevalent.

## 1 Introduction

### 1.1 Motivation

Dengue is a viral infection primarily transmitted by the bite of infected female *Aedes aegypti*. This mosquito has adapted to urban habitats in tropical and subtropical climates, often breeding in human-made water storage containers without secure lids or other objects that can accumulate rainwater such as buckets or tyres. Eggs can remain viable for months in dry conditions and hatch once in contact with water. Most cases are mild or asymptomatic, and medical intervention for severe dengue can lower fatality rates to below 1% (World Health Organization, 2023). However, in endemic areas, seasonal epidemics of varying size and intensity can overwhelm health service capacity, and so reliable dengue forecasts can inform mitigation efforts and reduce out-break severity.

In the past two decades, several models have been developed to account for factors influencing dengue transmission, some of which explicitly account for the effects of meteorological factors on vector population dynamics, and integrate data from entomological surveys with disease data (LaCon et al., 2014). In Vietnam, D-MOSS is the only currently available operational model that forecasts dengue cases (Colón-González et al., 2021). D-MOSS provides valuable province-level forecasts, taking historical earth observation data from MODIS and seasonal climate forecasts from the UK Met Office Global Seasonal Forecasting System version 5 (GloSea5) as inputs. Few tools have attempted to model the impact of interventions and make more robust estimates of the causal link between climate and infectious diseases (Colón-González et al., 2021; Reich et al., 2019). Dengue Advanced Readiness Tools (DART), funded by the Wellcome Trust, is a new project to develop a dengue outbreak warning system in Vietnam (University of Oxford, 2023). Inputs will include 10-to 14-day weather forecasts and higher-resolution disease forecasts will be produced at the district scale, bringing together multidisciplinary experts from the University of Oxford, the University of Science and Technology of Hanoi, and Vietnam’s National Institute of Hygiene and Epidemiology.

Given the well-established links of vector-borne diseases like dengue to environmental variation, weather variables, with their influence on vector population dynamics, behaviour and pathogen transmission (Colón-González et al., 2021; Do et al., 2014), are vital inputs to the DART system. Temperature, rainfall and changing land use affect the habitat availability of *Aedes aegypti* mosquitos (Kraemer et al., 2015), and extreme weather and climate change can influence the size and duration of outbreaks, often non-linearly (Nosrat et al., 2021). Incidence tends to lag the seasonal peak in rainfall and mean temperatures by 1–2 months (Do et al., 2014), which implies that skilful, localized forecasts able to predict possible spikes in disease transmission before they occur could prove immensely useful for healthcare planning.

An important initial step in the development of DART’s dengue early warning system is evaluation of the quality of meteorological inputs from the European Centre for Medium-Range Weather Forecasts (ECMWF) over Vietnam. As DART aims to provide district-level dengue forecasts by developing epidemiological models that use weather forecast variables as input data, it is crucial that the skill of the ECMWF forecasts over Vietnam is understood and assessed.

Verification of ECMWF outputs is currently focused on the extra-tropics (Owens & Hewson, 2018): ensemble forecasts of daily accumulated precipitation are skilful up to around a week ahead and 2-m temperature forecasts are known to retain skill after 9-day lead times (Haiden et al., 2022). Continuous improvements by ECMWF since the 1980s have seen substantial increase in extratropical skill (Magnusson & Källén, 2013). Post-processed ECMWF forecasts have shown skill compared with climatology for accumulated precipitation over grid points in tropical Asia, including Vietnam, at 5-day lead times (Vogel et al., 2020). The advent of predictability from ECMWF’s Ensemble Prediction System (EPS) for an extreme rainfall event in northeast Vietnam was around 3 days beforehand (van der Linden et al., 2017). However, skill over Vietnam at regional or city-specific scales relevant to dengue modelling in urban areas remains to be established.

### 1.2 Background

Vietnam runs 1650 km north to south from the Tropic of Cancer to around 8° N. The whole country is extremely thin, only 50 km wide at its narrowest point and 600 km at its widest, with much of the population living in close proximity to its extensive coastline. Around 70% of Vietnam’s precipitation falls in a rainy season from May to October. Peak precipitation is July–August in the North, while the South experiences almost constant heavy rain from June to October (Acharya & Bennett, 2021). Hanoi, Vietnam’s capital, is near the Red River’s delta within the otherwise generally mountainous North. It has a monsoon-influenced humid subtropical climate, with wet summers and relatively colder and drier winters (Kottek et al., 2006). Ho Chi Minh City is the largest and most populous city, and lies near the Mekong River’s delta in the South. It has a tropical monsoon climate, characterized by distinct transitions in April/May and October/November between the two seasons: wet/rainy and dry (Quan et al., 2021).

Vietnam’s atmospheric circulation is part of the Southeast Asian monsoon circulation and is also closely associated with the South Asian monsoon in summer, and the Northeast Asian monsoon in winter (United Nations Environment Programme, 2009). The Southeast Asian monsoon is a seasonal phenomenon, characterized by the northward shift of the intertropical convergence zone (ITCZ) and a reversal of prevailing winds during the rainy summer season. The ITCZ shift is caused by differential heating of the landmass and oceans in the summer months (driven by the ocean’s higher heat capacity and greater ability to mix heat away from the surface (Vreugdenhil & Gayen, 2021)). Increased temperature over land leads to ascent, and advection of moist air from the warm tropical Indian and Pacific Oceans. When this air, in turn, rises and cools, excess moisture is precipitated out as convective rain.

The heavy precipitation resulting from monsoon circulation is subject to complex interactions between land, ocean, atmosphere and solar radiative forcing (Bombardi et al., 2020), with both the South Asian—southwesterlies from the Bay of Bengal—and East Asian—southeasterlies from the South China Sea—monsoons affecting the dynamics (Zhang et al., 2002). Vietnam’s circulation in the north is also influenced by the subtropical and extratropical circulations. These processes together with the diverse local topography and considerable latitudinal range drive the variability of dry and rainy seasons experienced across Vietnam.

Dengue health burden in Vietnam shows strong regional and seasonal variation. Dengue is hyperendemic in two-season South Vietnam, with generally higher transmission in the rainy season. In northern Vietnam, with its four-season subtropical climate, dengue has been emerging for the past 20 years (Cuong et al., 2011). Local transmission peaks in the autumn (September to November) and is interrupted during the winter months, with outbreak severity varying interannually (Cuong et al., 2011; Cuong et al., 2013; Do et al., 2014; Rabaa et al., 2013; Thai et al., 2010).

Higher temperatures are known to increase the virus replication rate, shorten the extrinsic incubation period (the time after initial mosquito infection that dengue becomes transmissible to hosts), increase biting rates and increase the mosquito development rate by affecting the reproductive cycle, ovarian development and eclosion (emergence of adult mosquitos) of vectors (Cheng et al., 2020; Morin et al., 2013). However, sustained extreme temperatures, such as those experienced during heat waves, can suppress virus replication, reduce mosquito activity and even reduce the lifespan—and hence infectious potential—of vectors (Thai & Anders, 2011).

Precipitation plays a key role in the creation and maintenance of habitats essential for the mosquito life cycle (Morin et al., 2013). However, extremely heavy rain can flush away breeding sites (Colón-González et al., 2021). Much of the influence of meteorological variables on disease transmission depends on complex interrelations between meteorological, environmental, biological and sociodemographic factors. Vectors are limited in their range: it is the movement of human populations that is more likely to promote epidemic spread across regions. Favourable climatic conditions are required for disease transmission but are not sufficient without enough susceptible human hosts; lower population densities and higher immunity rates (exposure to one of the four major serotypes typically offers limited immunity to the other three) will tend to suppress transmission even in the presence of abundant infected vectors (Thai & Anders, 2011).

### 1.3 Ensemble forecasting

Probabilistic forecasts come from ensemble numerical weather prediction (NWP) systems, where equations from geophysical fluid dynamics governing the evolution of the atmosphere and oceans are solved and numerically integrated forward in time to estimate future conditions. ECMWF routinely runs global ensemble forecasts and reforecasts. Re-forecasts, also known as retrospective forecasts or hindcasts, are forecasts of the past made using up-to-date models and historical observations but without information considered to be from the future relative to the forecast date: this mimics forecasting with the benefit of being able to compare predictions with observations. The terms forecast and re-forecast are used interchangeably in this study, as both arise from the same ECMWF Integrated Forecasting System (IFS).

Forecasts are subject to inevitable uncertainty from errors in initial conditions and model formulations that grow in time (Palmer, 2000). In particular, where processes cannot be explicitly resolved in models, they are parameterized by relating them larger scale to variables that are resolved. Such parameterizations introduce additional errors and uncertainties that are accounted for through stochastic parameterization approaches. Non-linearity can lead to chaotic behaviour, with flowdependent high sensitivity to initial conditions where trajectories with small differences in their starting points rapidly diverge. The initial state is constrained by observations; perturbing both the initial state and model creates an ensemble of predictions that aims to characterize the uncertainty with a range of plausible values for each grid point.

Despite non-linearities, atmospheric circulation can “exhibit a degree of organization” on timescales of around 10 days (Palmer, 2000) and large-scale modes of variability with longer intrinsic timescales such as the El Niño–Southern Oscillation (ENSO) and Madden–Julian Oscillation (MJO) can contribute to predictability at lead times of several weeks. This suggests that forecasts could remain skilful at lead times beyond the short range in models where the relevant weather dynamics are well-characterized and when local-scale behaviour is largely dominated by large-scale influences on convection (Bombardi et al., 2020). Weaknesses in tropical forecasts from NWP systems can arise from both imperfect parameterization of convective processes that predominate and data assimilation systems designed primarily for the extra-tropics and drawing on comparatively fewer observations from tropical regions. Difficulties with the parameterization of small-scale processes like cloud microphysics, with potential to influence larger-scale conditions, may also be relevant (Vogel et al., 2020).

### 1.4 Aims and overview

This research aims to assess the skill of medium-range ensemble re-forecasts over the past two decades, contextualized by the prospect of using ECMWF inputs for a dengue early warning system. The rainy season (defined to be May–October inclusive) is the focus of this study, when dengue cases tend to peak in both North and South Vietnam and when monsoon circulation may provide reason to believe skill persists beyond a week. Re-forecast skill for 00Z 2-m temperature and daily total precipitation is assessed for lead times of up to 10 days. Two-metre temperature is the most relevant temperature variable to vector biology and disease transmission; varying the choice of the verification time from 00Z was subsequently found to not significantly alter results. 00Z corresponds to 7 am local Vietnamese time—slightly after dawn when, along with dusk, mosquitos tend to be more active in seeking hosts.

In the next two sections, the data, metrics for assessing skill, and methodology choices are covered. Section 4 details the results and provides discussion of their relevance, starting with an overview of spatial and temporal patterns in the reanalysis data and proceeding to the effect of bias correction on temperature re-forecasts. This understanding is used to interpret the forecast skill up to 10 days after initialization at different spatial scales and locations. Hanoi and Ho Chi Minh City (HCMC), and the larger areas around them, are used as exemplars, typifying the differences between North and South. There is also consideration of how independent observations affect assessment of precipitation skill. Finally, conclusions are presented in the last section with recommendations for further work.

## 2 Data

### 2.1 Observation data

#### 2.1.1 ERA5 reanalysis

ERA5 is ECMWF’s fifth-generation reanalysis, combining observations with short-range forecasts for a best estimate of the atmosphere’s state at a given time. This produces datasets that are temporally consistent, spatially complete and global in coverage (Hersbach et al., 2020). The high-resolution deterministic reanalysis is made available on a 0.25° grid (around 28-km resolution over Vietnam) and the domain used in this study covers latitudes 5–25° N and longitudes 100–110° E. Information about the observational datasets used in this study can be found in Table 1. ERA5 assimilates a wide range of global satellite and ground-based observations, including surface temperature and precipitation.

Precipitation data are calculated as the total daily precipitation accumulated during the 24 h following 00Z (UTC + 0); cumulative precipitation over multiple days is calculated by summing the daily precipitation for the relevant time period. Temperature data take the form of 2-m temperature (i.e. roughly the temperature experienced by mosquitos just above the surface during their lifecycle) at 00Z, which is 7 am local time in Vietnam.

ERA5 is used as verification data due to its spatial and temporal completeness. With a combination of assimilated observations and model physics, it allows a complex state estimation of observed and non-observed variables beyond their individual availability.

#### 2.1.2 VnGP

The Vietnam Gridded Precipitation (VnGP) dataset was constructed using the Spheremap interpolation technique and daily rainfall (accumulated in the 24 h since 12Z the previous day) recorded at 481 rain gauges across Vietnam (Nguyen-Xuan et al., 2016). VnGP data at 0.1° spatial resolution for the period 1980–2010 were extended to 2018 with data from 189 gauges. The updated data for 2010–2018 were provided for use in this study (personal communication). Another VnGP dataset at 0.25° up to 2007 has been verified with independent observations (Nguyen-Xuan et al., 2016), lending some confidence in the interpolation technique used. The station locations are available on a map from the CPIS Vietnam website (CPIS, 2019).

Unlike the ERA5 reanalysis, VnGP data are independent of ECMWF’s dynamic model and are based on deterministic interpolation of observations from Vietnam alone. They have not been assimilated into ERA5. The nature of convective precipitation in tropical climates means localized rain gauge recordings could differ significantly from gridded ERA5 values. Verification with VnGP data is intended to give an indication of whether skill is notably different when based on independent rain gauge measurements in Vietnam, although it must be noted that these data are themselves an interpolation and are not necessarily always representative of local rainfall totals.

There is a 12-h offset in the start of the accumulation time compared with ECMWF precipitation outputs and data points only align at latitudes and longitudes ending in 0.0 or 0.5°. When comparing VnGP to re-forecasts, it is necessary to match spatial boundaries (see Section 3.1 and Table S1) to the 0.1° resolution: 0.25° is truncated down to 0.2° and 0.75° rounded up to 0.8°. Varying the choice of these boundaries does not produce significantly different results. The imperfect spatial and temporal alignment between re-forecast and VnGP data are further grounds for initially focusing on verification with ERA5.

### 2.2 Re-forecast data

ECMWF ensemble predictions include state-of-the-art atmospheric, land surface, oceanic and sea-ice components. Full details of the model physics can be found in the IFS Documentation Cy47r3 (ECMWF, 2021a). Forecasts always come with a 20-year set of re-forecasts for calibration and skill assessment. The re-forecasts analysed in this study used model version Cy47r3. They have horizontal atmospheric resolution of T_Co639_, with 91 vertical levels. The ocean model is the Nucleus for European Modelling of the Ocean (NEMO) version 3.4.1 in the ORCA025 configuration: 0.25° horizontal resolution, with 75 vertical levels. Initial conditions are from the ERA5 reanalysis (see Section 2.1.1).

The re-forecasts used in this study were initialized on consistent dates twice a week every year (corresponding to every Monday and Thursday in 2021). The first initialization date is 04/01/2001 and the last date is 30/12/2020. The ensemble is made up of an unperturbed control forecast and 10 perturbed ensemble members (ECMWF, 2021b), which together create the 11-member re-forecast ensemble used here (see also table 1b in ECMWF’s *Operational configurations of the Integrated Forecasting System* (2023)). As with ERA5, the data are available on a 0.25° grid (around 28-km grid-point resolution). Table 2 summarizes the forecast datasets used.

Accumulated precipitation is forecast from the initialization time (00Z on the initialization date) to a given end time. Accumulation times vary from 0 to 10 days at 6-hour intervals, although the focus for this study is 1-day (24-h) intervals. The total daily precipitation is calculated by subtracting from the accumulated precipitation the total to the end of the previous day. An *x*-day lead time is taken to mean a forecast of the total daily precipitation initialized *x* days before the end of the day.

When comparing against VnGP, total daily precipitation is taken to be from 12Z on the forecast date to 12Z on the following day. This necessarily adds an additional 12 h to each lead time, as forecasts are still initialized at 00Z on the initialization date.

As with the reanalysis, the 2-m temperature re-forecast values are daily at 00Z. An *x*-day lead time forecast is made 24 × *x* hours beforehand and can be compared directly with the corresponding reanalysis value.

The re-forecasts are accompanied by orographic data of the topography in Vietnam, on the same grid as the reforecasts. Details can be found in Table 3.

## 3 Methods

### 3.1 Study area

**Figure 1:**
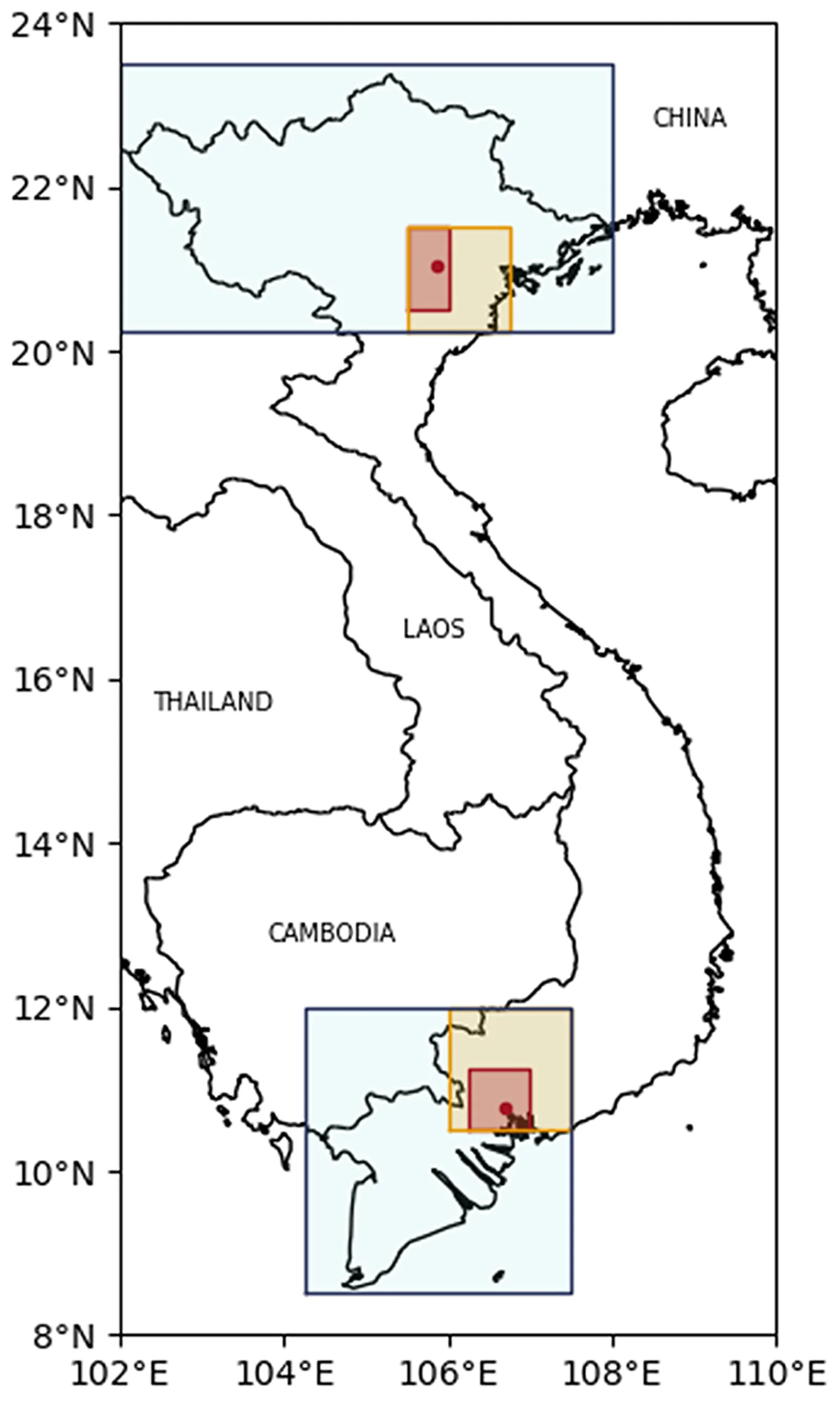
The spatial scales for which forecasts are considered. Blue boxes show North and South Vietnam, gold boxes show regions and red boxes are municipalities around the two cities (shown as dots). Refer to Table S1 for further information

Hanoi and HCMC are the exemplar cities chosen by the DART project and hence the focus areas for this work. The 0.25° resolution of both the re-forecast and reanalysis data prevents exploration of skill at the scale of a few kilometres, but the intention for this research is to understand whether ECMWF re-forecasts have skill more generally over municipal and regional scales in Vietnam. A rectangle bounding Vietnam is the largest spatial scale considered, with latitudes 8–24° N and longitudes 102– 110° E. Spatial scales are bounded with values in Table S1 and can be seen relative to each other in Figure 1. The city scales are determined by bounding boxes around their respective municipalities, while the region boundaries are determined by bounding boxes for the Red River Delta and Southeast regions within which the cities lie. These study areas were chosen to explore how skill varies with spatial scale and between the North and South. It should be noted that North Vietnam and South Vietnam in this study refer to specific bounding boxes that include parts of land and sea beyond Vietnam’s borders, rather than the north and south of the country more generally.

### 3.2 Bias correction

Quantile mapping (QM) is a common way to correct for systematic bias in forecasts (Cannon et al., 2015)—it is performed for temperature forecasts at each grid point in North and South Vietnam using re-forecast and ERA5 reanalysis data. For a given year and month, ERA5 cumulative distribution function (CDF) *G*, re-forecast CDF *F* and forecast value $\hat{x}$, the corrected value calculated as

(1) $$\hat{x}=G^{-1}[F\{x\}].$$

**Figure 2:**
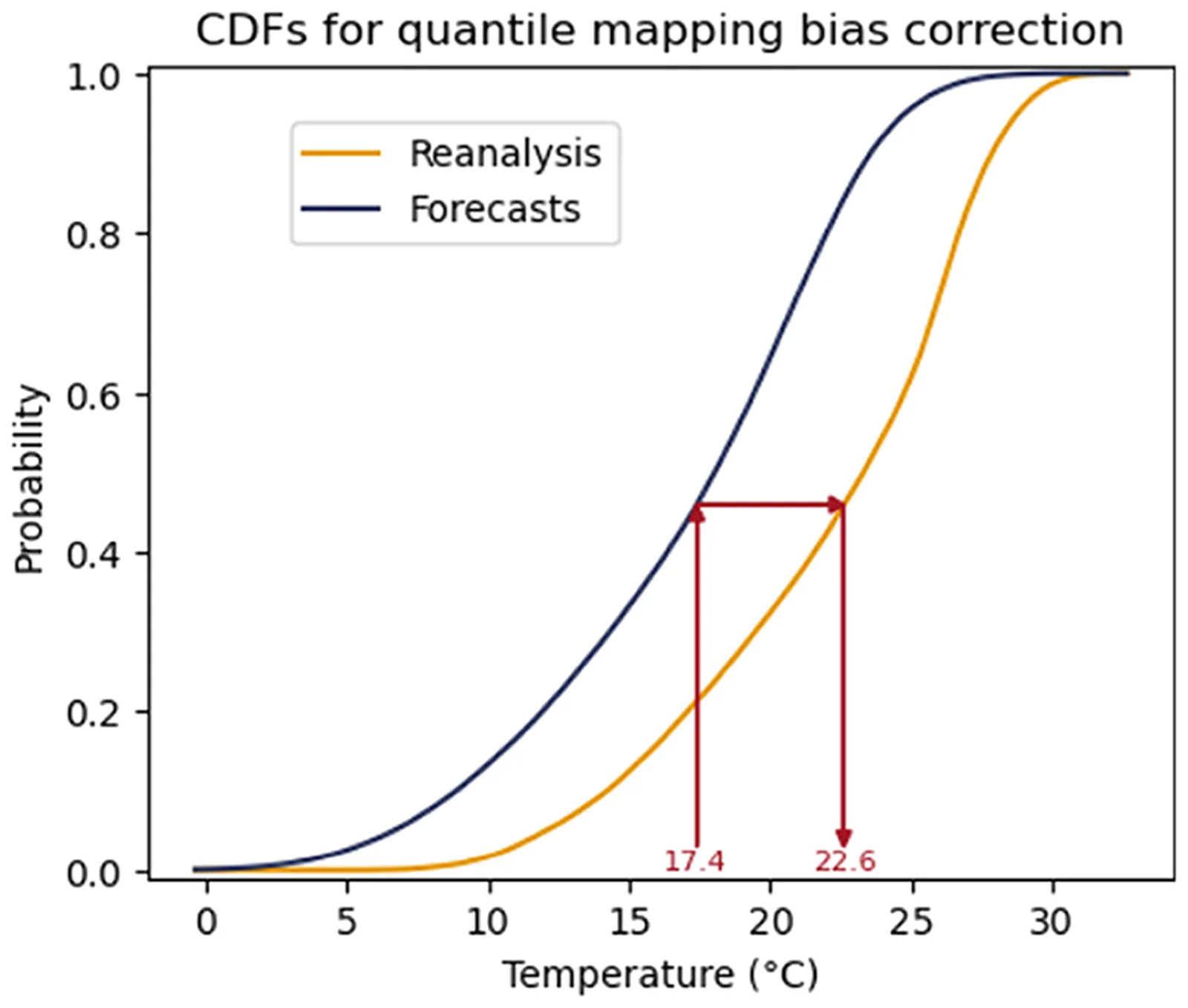
Schematic to show the process of correcting a systematic cold bias with quantile mapping using the cumulative distribution functions (CDFs), for example reanalysis and forecast data.

To avoid artificial skill (Jolliffe & Stephenson, 2011), the corrected value uses the CDFs for years excluding the target year (cross-validation mode). The QM process is visualized in Figure 2: the probability of the random variable not exceeding the forecast value is found with the re-forecast CDF and the value corresponding to that probability with the ERA5 CDF becomes the corrected $\hat{x}.$ Individual months are corrected separately, with reforecast CDFs created using the month the prediction was made, for each latitude–longitude pair. As ERA5 and the re-forecasts are not fully independent, bias arising from the underlying model may not be corrected for by this process.

### 3.3 Metrics

The natural choices for ensemble forecasting of a continuous variable are probabilistic, continuous metrics. All metrics used for forecast verification have strengths and weaknesses. In many cases, it is useful to compare a forecast to a performance baseline. This study uses both persistence and climatology as reference forecasts to benchmark skill. A forecast is considered ‘skilful’ relative to persistence if it can outperform a distribution based on the previous day’s value; it is ‘skilful’ relative to climatology if it outperforms a distribution formed from observations of previous years.

The 11-member persistence ‘ensemble’ is constructed using the value the day before a re-forecast is made and the standard deviation of the re-forecast ensemble at that location and lead time. Members are randomly sampled, assuming a normal distribution for temperature and a log-normal distribution for precipitation (Cho et al., 2004; Kedem & Chiu, 1987; Ng et al., 2018; Wilks, 2011). In the case of cumulative persistence, each of the persistence values is itself the total of *x* days of precipitation ending on the day before the forecast was initialized. The climatology ‘ensemble’ is also constructed for each grid point and time, with three values from each year (the day itself and 1 day on either side) selected for every year of available data, excluding the target year (cross-validation mode).

This study uses lead times of up to 10 days to assess the short- and medium-range skill of ECMWF ensembles. Persistence can be an informative reference forecast for assessing short-range skill (Jolliffe & Stephenson, 2011). Temporal autocorrelation is useful for understanding how forecast skill compares to persistence and is calculated for the rainy season by averaging the value for each calendar date over the years of available data. It is particularly appropriate to tropical climates, where conditions can demonstrate persistence up to and beyond the medium-range, potentially as a result of monsoon circulation (Bombardi et al., 2020). The data are correlated with a lagged version of itself at each grid point to obtain autocorrelation for that given location. To calculate a value for each area, the mean within the area is taken. In the absence of a forecast system or climatological distribution to draw upon, an a priori approach to dengue prediction might reasonably use the most recent weather conditions as input, hence the need to show skill relative to persistence. As lead times increase, climatology would be assumed to be the more competitive reference.

#### 3.3.1 Rank histograms

For a perfect forecast, the ensemble members should represent a random sample from the underlying distribution that quantifies model uncertainty. As such, the verifying observation should be statistically indistinguishable from the ensemble (Wilks, 2011). For *N* forecast–observation instances and *M* ensemble members, the rank of the observation within the *M* + 1 values is calculated and the frequency of taking each rank is plotted on a histogram. In a reliable forecasting system, there should be uniformity in rank. Reliability in this context refers to the conditional relationships between forecast and observation values, that is, relationships for given forecast values. Reliable systems do not require re-calibration to remove bias (Jolliffe & Stephenson, 2011).

The extent to which histogram values deviate from *N*/(*M* + 1) elucidates weaknesses: too few instances of populating the first and last ranks suggest an overdispersive ensemble, whereas under-population of central ranks implies overconfidence and that the ensemble is not effectively capturing the full spectrum of observed values. Similarly, overpopulation of lower ranks is suggestive of overforecasting (consistently overestimating the value), and vice versa.

Rank histograms are tabulated using forecast– observation pairs (*M* + 1 = 12) at each grid point within the area of interest and for each day within the rainy season (May–October) over the whole 2001–2020 period of the re-forecast data. A chi-squared test is typically used to assess the statistical significance of deviation from flatness. However, this would not be meaningful in the given circumstances for two reasons: the *N* instances are not independent (Wilks, 2011), as there is significant spatial and temporal autocorrelation; and the value of *N* is large enough that all *p*-values are extremely small.

#### 3.3.2 CRPSS

The continuous ranked probability score (CRPS) is a common continuous and probabilistic metric for evaluating forecasts, allowing assessment of skill over the full range of temperature or precipitation values experienced and for the whole ensemble (Hersbach, 2000; Matheson & Winkler, 1976; Mishra et al., 2019). Where *y* is the observed value, *H* is the Heaviside function with value 0 when *x* < *y* and *F* is the cumulative density function of ensemble forecasts,

(2) $$\text{CRPS}=\int_{-\infty }^{\infty }{\left[F(x)-H(x-y)\right]}^{2}dx.$$

CRPS might be considered a probabilistic generalization of the mean absolute error (MAE) score, and can also be calculated as the integral over all possible thresholds of the commonplace Brier score (Hersbach, 2000), permitting consideration of the whole distribution range. The continuous ranked probability skill score (CRPSS) gauges the CRPS against a reference forecast—either climatology or persistence in this case—and is a positively-orientated measure of skill, where 1 indicates perfect skill and 0 indicates a forecast no better than the reference. The reference forecast CRPS is calculated using the reference ensemble in place of the forecasts such that

(3) $$\text{CRPSS}=1-\frac{\text{CRPS}}{{\text{CRPS}}_{\text{ref}}}.$$

The CRPSS can be calculated for both daily and cumulative values for different lead times. When assessing skill, it is more informative to separately compute scores for each subset of the data to generate a distribution, rather than generate a single score from aggregated data (Jolliffe & Stephenson, 2011). By calculating the skill score for each grid point, a given area can be characterized by its average spread and range of skill scores.

## 4 Results

### 4.1 Spatial and temporal patterns

**Figure 3:**
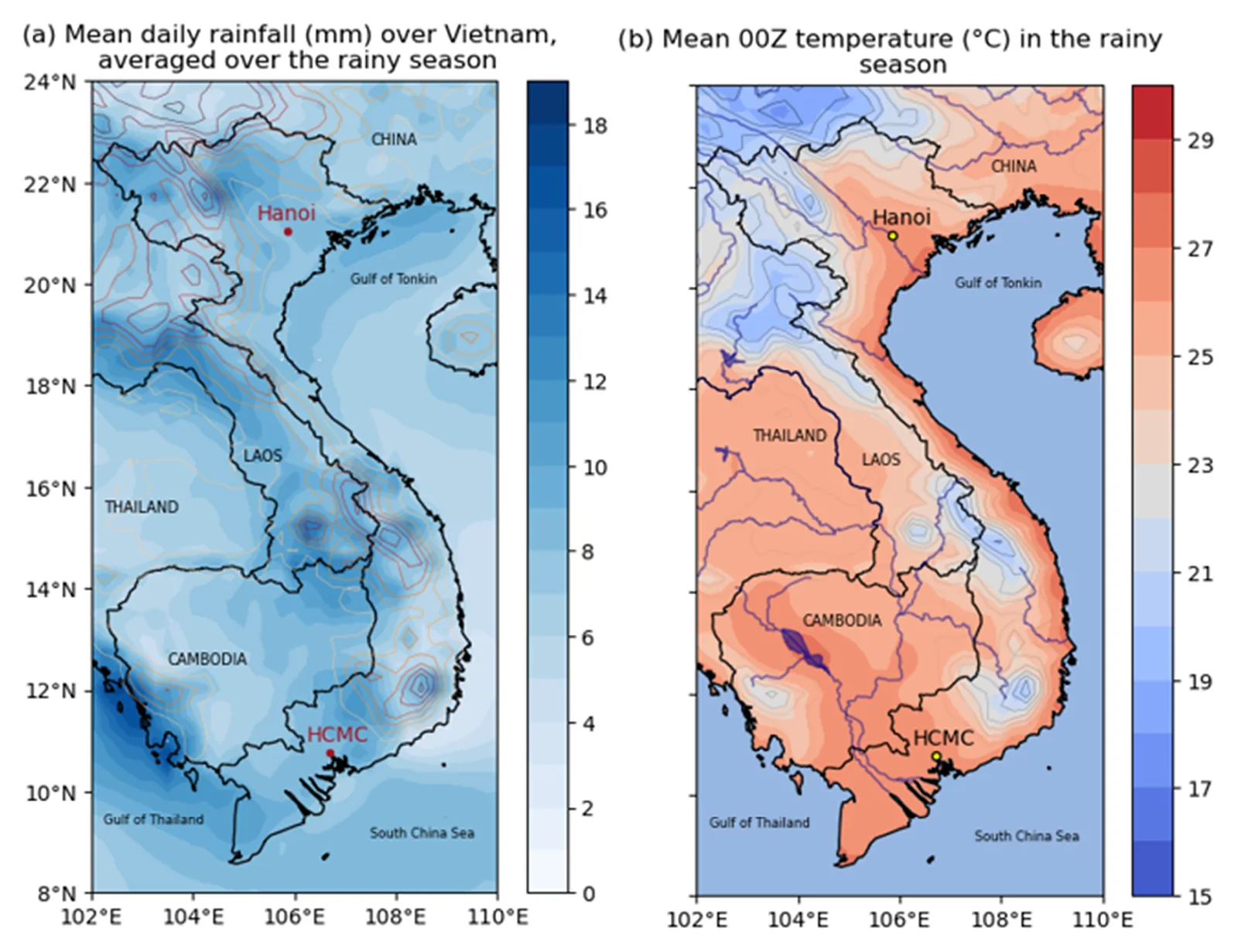
Maps of the rainy season (May–October) over Vietnam and the surrounding areas using ERA5 reanalysis data. Contour lines show geopotential height. (a) Mean total daily precipitation (2000–2022)—precipitation maxima are often close to orographic features. (b) Mean 00Z 2-m temperature (2001–2020)—higher in flatter areas towards the coast.

**Figure 4:**
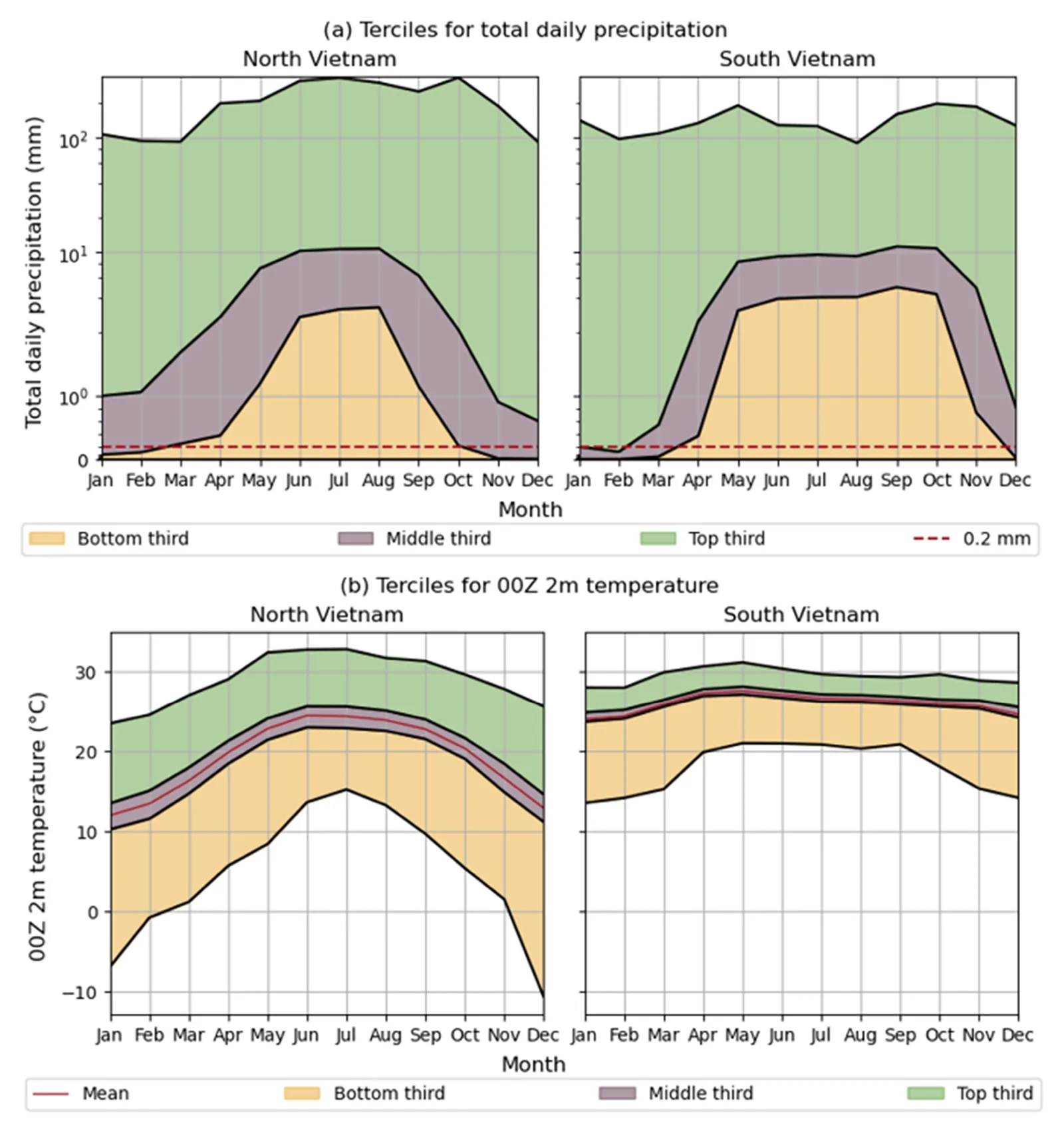
ERA5 terciles for each month in North and South Vietnam—a third of the data lies in each band. (a) Total daily precipitation (01/01/00 to 31/10/22). Note the log scale and the 0.2-mm line (a threshold for a day to be considered ‘dry’). The rainy season in the South has less gradual transition. (b) 00Z 2-m temperature (01/01/01 to 31/12/20). South Vietnam has notably less variation in temperature. Mean temperatures (shown in red) are most similar between North and South in the rainy season.

The ERA5 data match the known annual cycle for both temperature and precipitation in North and South Vietnam (Acharya & Bennett, 2021; Kottek et al., 2006; Nguyen et al., 2014; Quan et al., 2021; United Nations Environment Programme, 2009). Figure 3, showing mean daily precipitation and 00Z temperature respectively during the rainy season (May–October inclusive), indicates that precipitation maxima coincide with orographic features, while the highest mean temperatures tend to be in flatter areas in the south or towards the country’s east coast. The mean rainy season temperature is around 26–27°C in both Hanoi and HCMC. North Vietnam has a more defined annual cycle: temperature peaks in boreal summer (JJA) and has a minimum in boreal winter (DJF), with a change in mean temperature of around 10°C in Hanoi between winter and summer. South Vietnam has a much smaller annual cycle, with mean temperature varying by less than 5°C in HCMC. The range of typical rainy season daily precipitation is from a few millimetres to around 10 mm, although precipitation an order of magnitude greater (i.e. 100 mm or even above) is not unheard of. Figure 4 splits the ERA5 distributions of temperature and precipitation values into terciles and illustrates the difference between the more gradual onset and termination of the monsoon rainfall in North Vietnam compared with South Vietnam, which lacks an obvious transitional stage.

**Figure 5:**
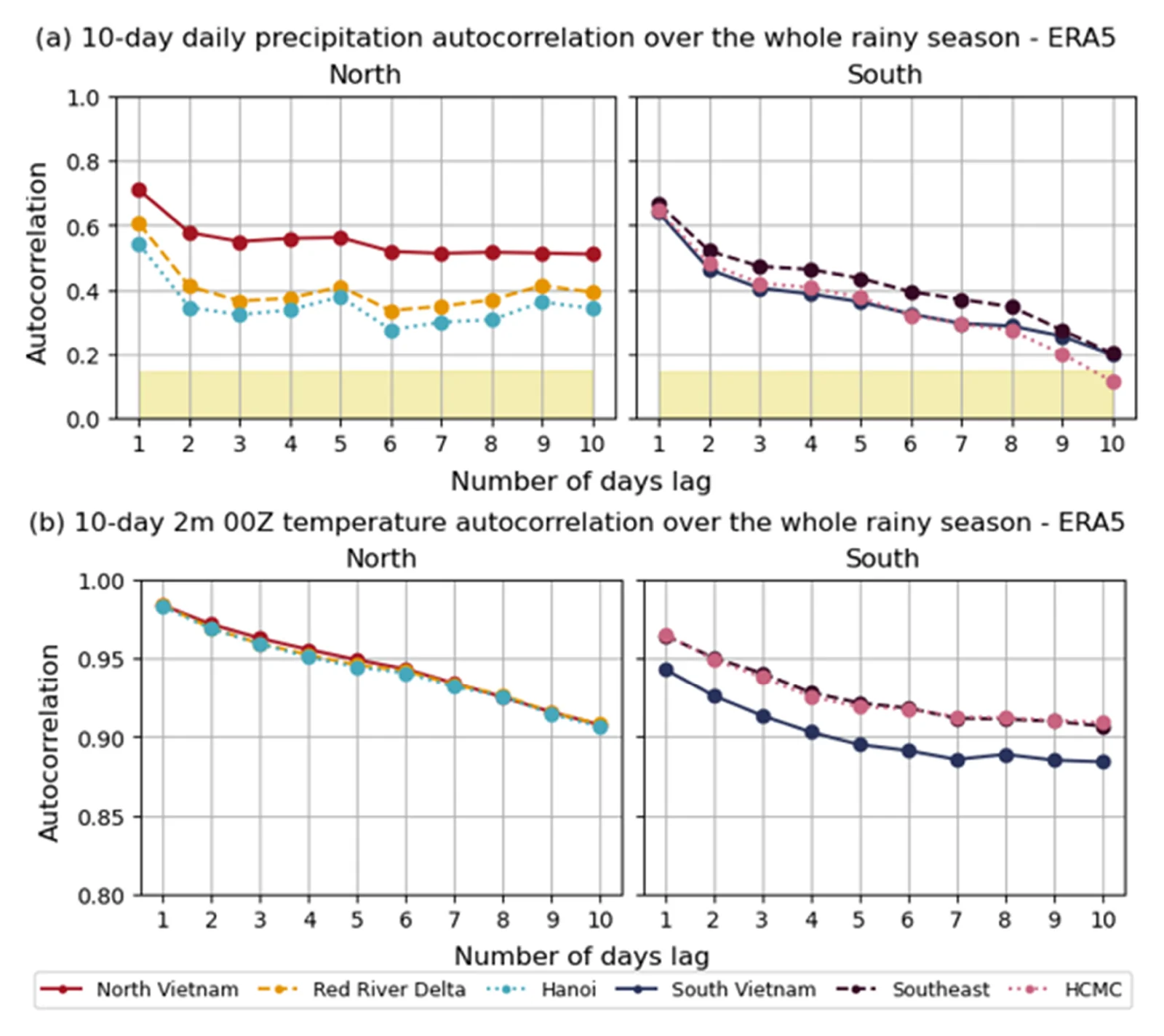
ERA5 temporal autocorrelation in the rainy season (May–October) for each location as lag increases up to 10 days. (a) Daily precipitation. The box shows the 95% confidence interval—values outside of this are considered statistically significant. Note that autocorrelation in the North remains significant after 10 days. (b) 00Z 2-m temperature. There is little difference between spatial scales in the North but macro-region autocorrelation is lower in the South overall than for the region and municipality around HCMC. Autocorrelation is much higher than for precipitation and hence the region for statistical significance is not shown.

Rainy season autocorrelation is high for both temperature and precipitation (Figure 5). Precipitation demonstrates differences in autocorrelation between North and South: unlike the North, the South’s autocorrelation is no longer significant after about a week. This may be explained by the differing tropical and subtropical climates in South and North Vietnam, with more variability introduced by tropical systems like the MJO in the South and the influence of the Western North Pacific Subtropical High promoting highly persistent conditions in the North. Temperature autocorrelation values are even higher than those for precipitation, only falling to around 0.9 after 10 days; this persistence of rainy season conditions justifies the exploration of persistence as a reference forecast.

### 4.2 Bias

**Figure 6:**
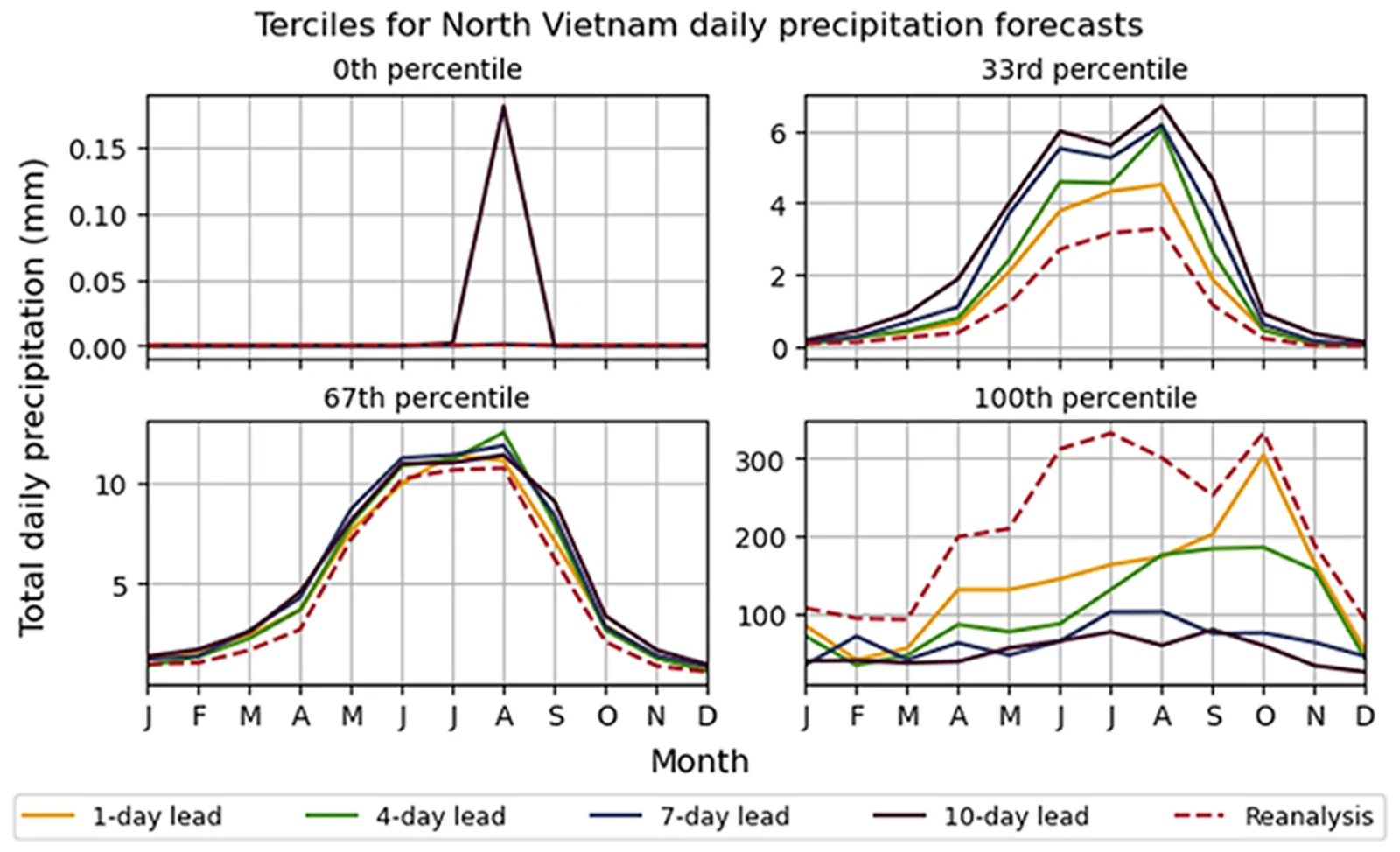
Comparison of percentiles demarcating the monthly terciles for total daily precipitation in North Vietnam for the re-forecasts and reanalysis (red). Compare with Figure 7 for South Vietnam and note the changes in scale and increasing difference to reanalysis as lead time increases.

**Figure 7:**
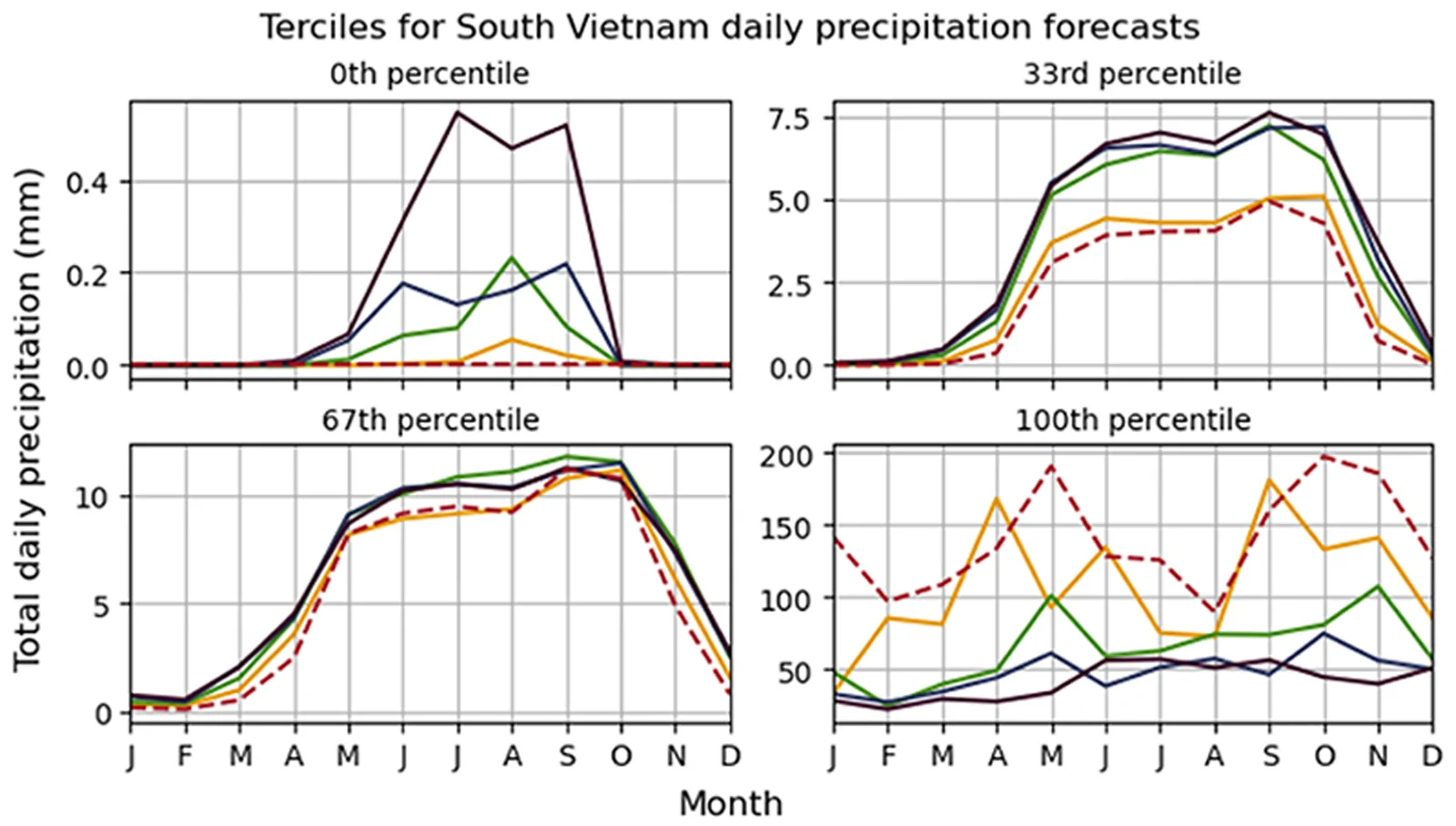
Comparison of percentiles demarcating the monthly terciles for total daily precipitation in South Vietnam for the re-forecasts and reanalysis (red). Compare with Figure 6 for North Vietnam and note the increase in rainy season 0th percentile as lead time increases.

Bias for a given grid point and observation time is simply calculated as the difference between the re-forecast and observation (ERA5) values. Figures 6 and 7 show the bias in precipitation re-forecasts for North and South Vietnam. Reanalysis, in red, is compared with re-forecasts at four different lead times for terciles of the reanalysis and reforecast distributions. The re-forecasts closely match the reanalysis at the 67th percentile in both locations throughout the annual cycle. They perform less well at the 0th and 100th percentiles, suggesting prediction of more extreme values is poorer and a tendency to overestimate precipitation when it is dry and underestimate it when it is very wet. Longer lead times are particularly deviant from the reanalysis in this respect, suggestive of good performance around the median values (middle tercile) but poorer performance when forecasting extremely heavy rain, or telling dry conditions from drizzle or short-lived, localized convective rain. Overprediction of drizzle in NWP models is well-documented (Ahlgrimm & Forbes, 2014; Stephens et al., 2010; Wilkinson et al., 2013). This is a particular issue in the South, where it is infrequently dry in the rainy season and the re-forecasts significantly overpredict the 0th percentile. The same bias patterns can be observed at regional and municipal scales (not shown).

**Figure 8:**
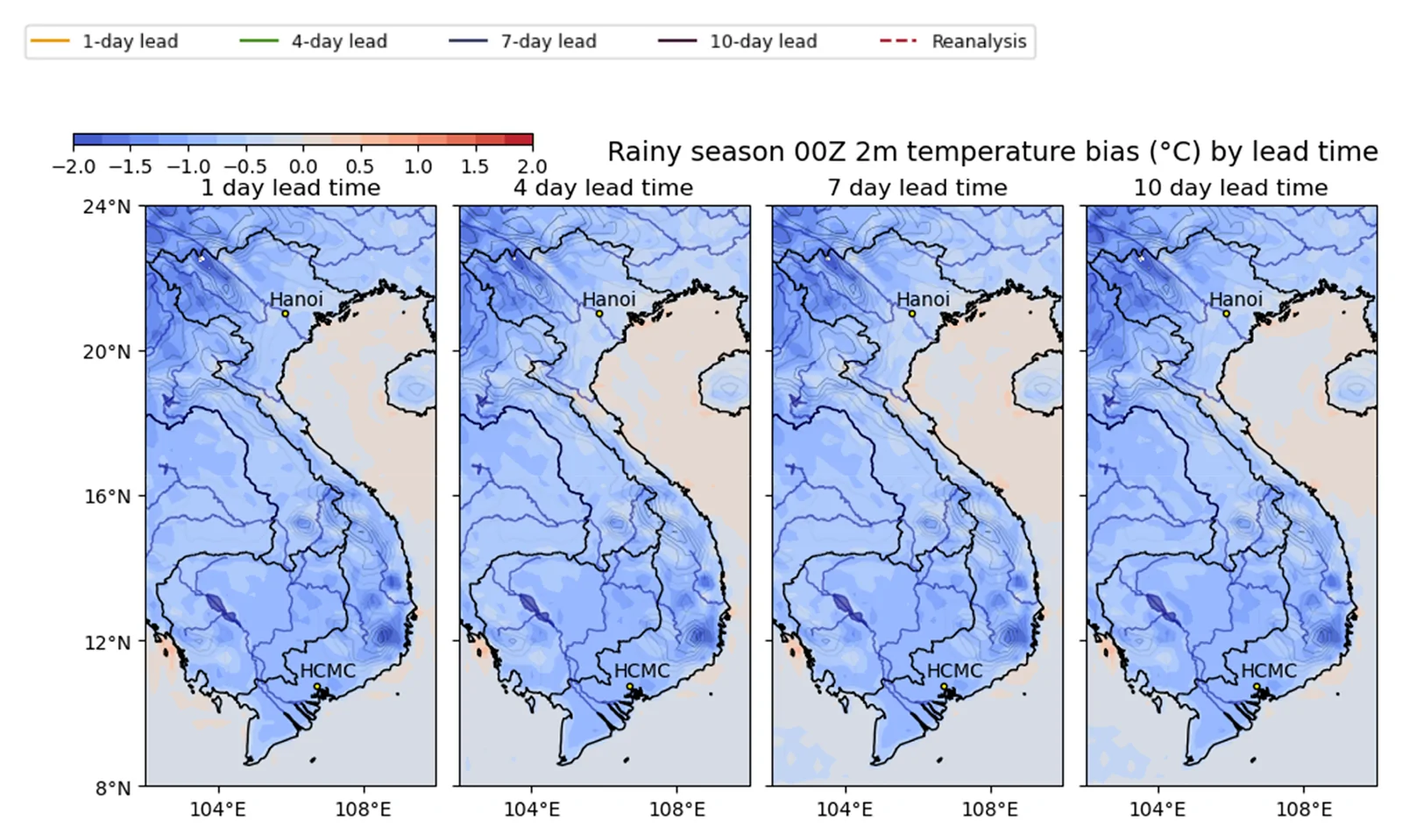
Maps of the cold bias (difference between re-forecast and ERA5 values for 2001–2020) in 00Z 2-m temperature over Vietnam during the rainy season (May–October) for lead times increasing up to 10 days. There is little difference in bias between lead times. The cold bias can be corrected for with quantile mapping.

Temperature, on the other hand, presents a consistent cold bias across spatial scales, locations, lead times and the annual cycle. The bias exists at 1-day lead times and shows no obvious growth or decay trend. This is consistent with cold biases known to exist in ECMWF outputs, including for areas of China not far from North Vietnam (Haiden et al., 2018; Hou et al., 2022; Wei et al., 2021). The mean rainy season cold bias (Figure 8) is up to around 1°C but generally around 0.5°C in the study areas. A comparable cold bias is also seen in the dry season (Figure S1). Temperature re-forecast distributions are otherwise similar in shape to those of the reanalysis. Data correction by QM is used to ameliorate the cold bias, having the effect of shifting temperature re-forecast distributions up to better match ERA5 (as can be seen comparing Figures S1 and S2).

### 4.3 Verification at varying lead times

**Figure 9:**
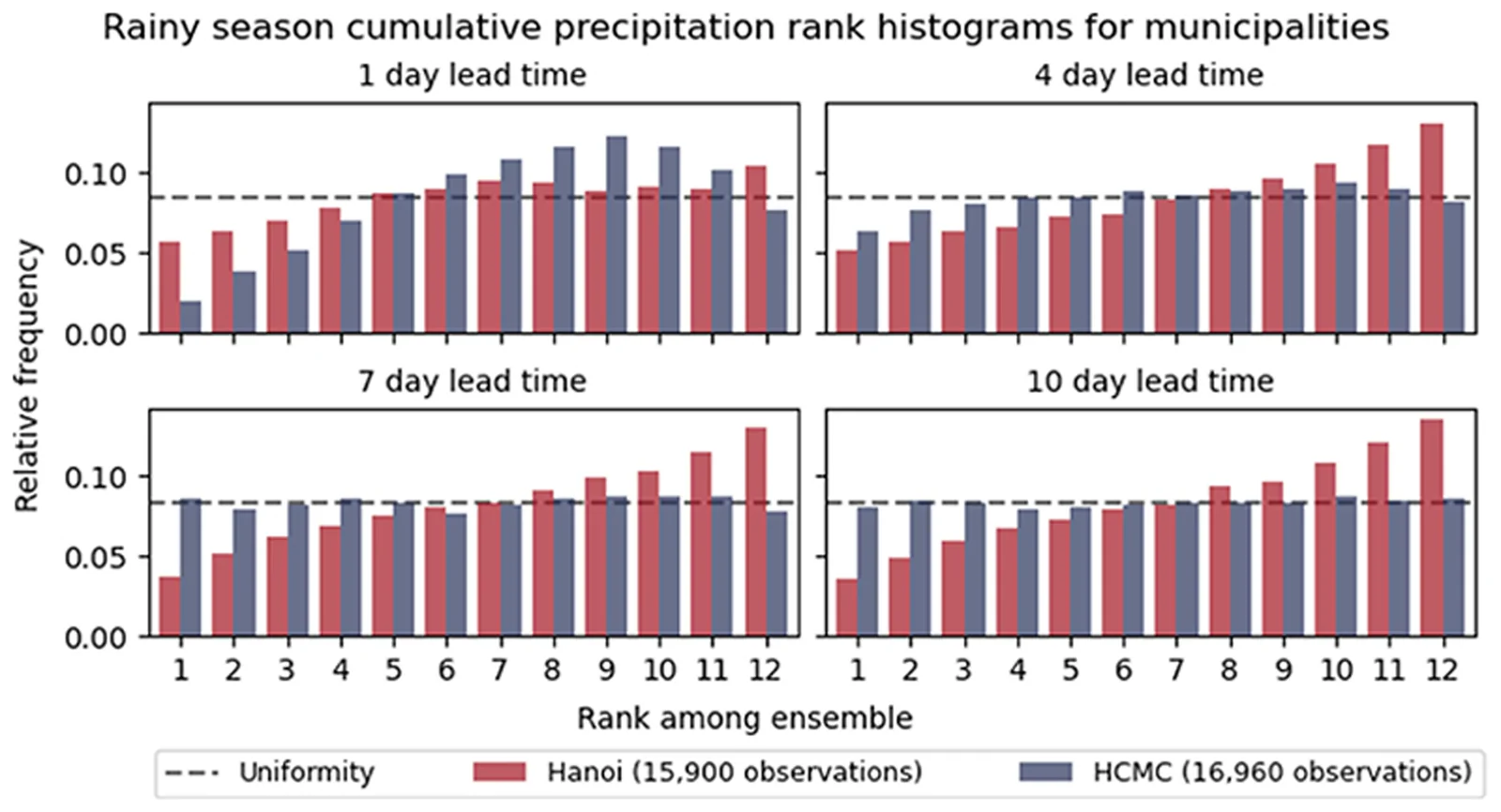
Rank histograms for rainy season (May–October) cumulative precipitation at 1-, 4-, 7- and 10-day lead times over Hanoi (red) and HCMC (blue). One-day forecasts over HCMC suggest underconfidence, whereas at longer lead times they are flatter than for Hanoi, which appears to show a degree of underforecasting.

Rank histograms for rainy season cumulative precipitation for the Hanoi (red) and HCMC (blue) municipalities are shown in Figure 9. Lead times of 4, 7 and 10 days over HCMC perform notably well, indicating significant forecast reliability. 1-day lead times over HCMC are more prone to underconfidence (the ensemble range is too large), and at longer lead times, there is increasing under-forecasting over Hanoi. Rank histograms at larger spatial scales are generally similar, and hence are not shown, although there is more tendency towards overforecasting for the South as a whole compared with the HCMC municipality.

**Figure 10:**
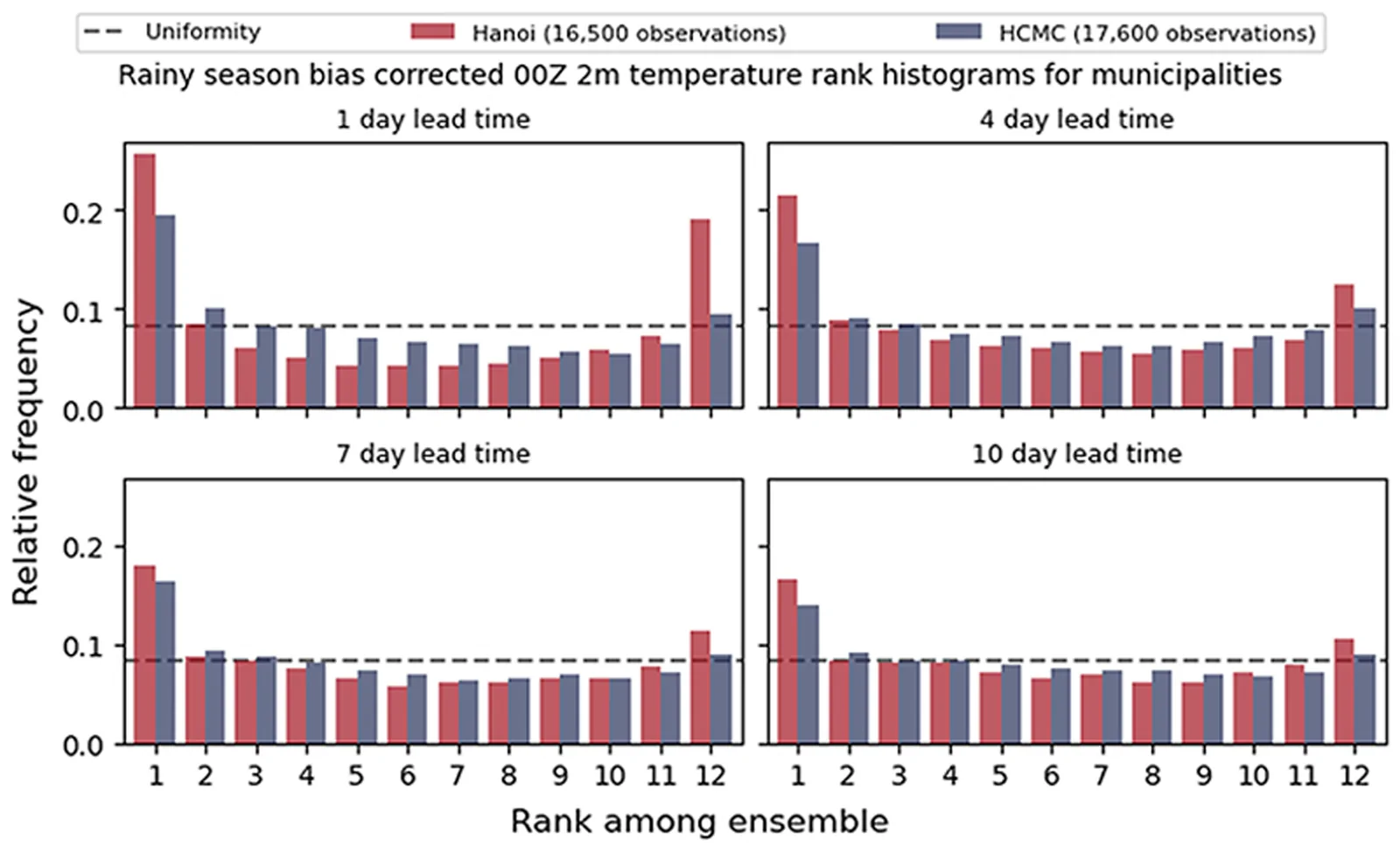
Rank histograms for rainy season (May–October) bias-corrected 00Z 2-m temperature at 1-, 4-, 7- and 10-day lead times over Hanoi (red) and HCMC (blue). The re-forecasts are overconfident, with overpopulation of the outer ranks, and more so over Hanoi than for HCMC. Uniformity also shows improvement with lead time.

00Z temperature rainy season rank histograms using uncorrected values (Figure S3) demonstrate the anticipated underestimate of temperature expected from a systematic cold bias, seen as an obvious overpopulation of the highest rank, particularly for HCMC. Underestimating the temperature is substantially corrected by QM (Figure 10). These histograms are generally closer to uniformity, showing good reliability that has positive implications for successful use of bias-corrected temperature in the DART system. Nevertheless, they display some degree of overconfidence, that is, the observed temperatures are typically either below or above the ensemble range. This overconfidence slowly decreases with lead time. This is true also for the larger spatial scales around the two cities (not shown).

**Figure 11:**
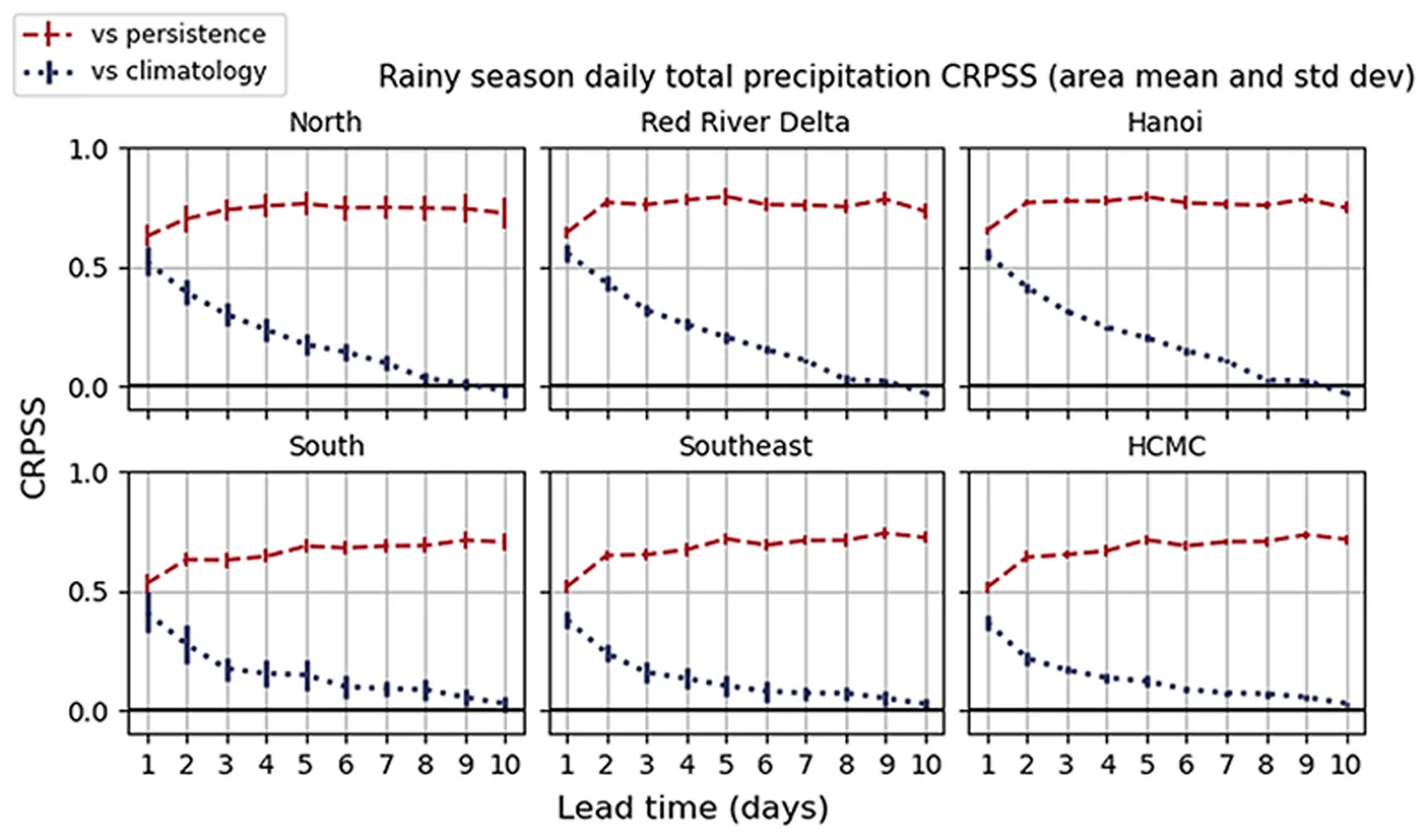
Rainy season (May–October) daily total precipitation CRPSS values for each location and scale with lead times up to 10 days, showing both persistence (red) and climatology (blue) as reference forecasts. In all locations, positive CRPSS values demonstrate that re-forecasts are overall significantly more skilful than both persistence and climatology. The uncertainty range (the spread indicated with error bars) is estimated with the root mean squared error, that is, the standard deviation. The uncertainty range of values for each area is smaller for the smaller spatial scales, likely because the smaller areas’ features are more homogeneous.

There is substantial and significant skill in predicting rainfall and temperatures out to 10 days when measured against both persistence and climatology as reference forecasts. Positive CRPSS values demonstrate that the ECMWF re-forecasts are significantly more skilful than both persistence and climatology. The CRPSS for daily precipitation against climatology (blue) shows a clear decline in skill with lead time from around 0.5 in the North and 0.4 in the South to just below (North) or above (South) zero after 10 days (Figure 11). Compared with the South, areas in the North tend to initially have greater skill but decline more rapidly with time. Re-forecasts benchmarked with climatology retain skill for just over a week in the North and for the whole 10-day period considered in the South.

Using persistence (red) as the reference forecast does not produce a decline in skill with lead time. After an increase to around 0.75 between 1- and 2-day lead times, scores remain generally unchanged for locations in the North besides a slight reduction for the 10th day. The South has a gradual increase in skill over the first 5 or so days before similarly levelling off to around 0.75. These differences may relate to the autocorrelation characteristics of the two macro-regions (Figure 5a), with generally higher autocorrelation in the North than South after 5 days of lag and a more gradual decline in the North after the initial drop between 1 and 2 days.

**Figure 12:**
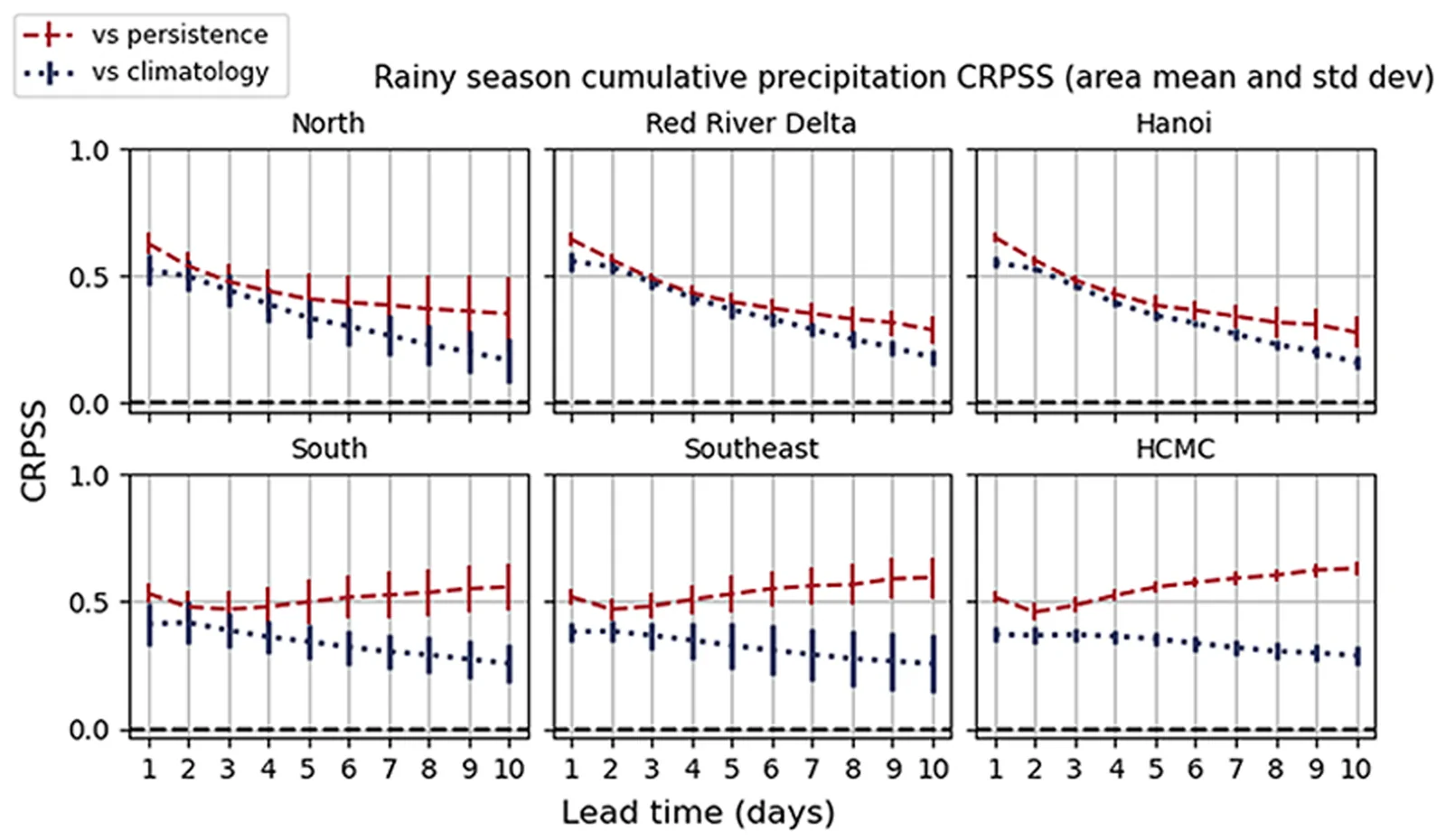
Rainy season (May–October) cumulative precipitation CRPSS values for each location and scale with lead times up to 10 days, showing both persistence (red) and climatology (blue) as reference forecasts. Skill compared with climatology is initially comparable to those for daily total precipitation values (Figure 11), but the decline with lead time is less steep, particularly in the South. Mean scores versus climatology remain above around 0.2 for all locations for lead times up to 10 days, and skill scores in the South are better at longer lead times than for the North. When persistence is used as the reference forecast, skill scores over the North show a similar decline to those compared with climatology, although persistence appears to generally be a weaker reference as scores are consistently higher. However, skill scores compared with persistence in the South show a decided increase with lead time after 2 days, again potentially related to the lower autocorrelation in the South, making persistence easier to beat as predictions move further into the future.

CRPSS for cumulative precipitation (Figure 12) compared with climatology (blue) shows less steep decline in skill over the 10 days. This suggests that cumulative precipitation outputs from re-forecasts with longer, subseasonal lead times may also be skilful. All six areas of interest continue to have scores above around 0.2 after 10 days. As with daily precipitation, scores in the North start above 0.5 and in the South just below, with a slower decline with lead time seen for the latter. Encouragingly, re-forecasts for smaller spatial scales (i.e. at municipality level) do not show significantly different skill to larger areas and indeed have a generally smaller range of scores, indicated by the standard deviation. The differences between decline with lead time in North and South Vietnam can also be seen in maps of skill scores in Figure S4. Longer accumulation times have previously demonstrated improved performance, especially in wetter tropical areas, due to less stringent requirements to predict the exact time of precipitation events (Vogel et al., 2020).

CRPSS compared with persistence (show in red in Figure 12) for cumulative precipitation demonstrates more similarity to values for climatology over North Vietnam than is seen for daily values. However, scores are consistently higher, indicating that persistence is the weaker reference forecast. Over South Vietnam, CRPSS with a persistence reference shows a marked increase with lead time after the second day, more so than for daily precipitation. Again, this likely relates to lower autocorrelation in the South, making persistence increasingly easy to beat as predictions are made further and further away in time. In all locations, the gap between CRPSS values with a climatology versus persistence reference grows with lead time.

**Figure 13:**
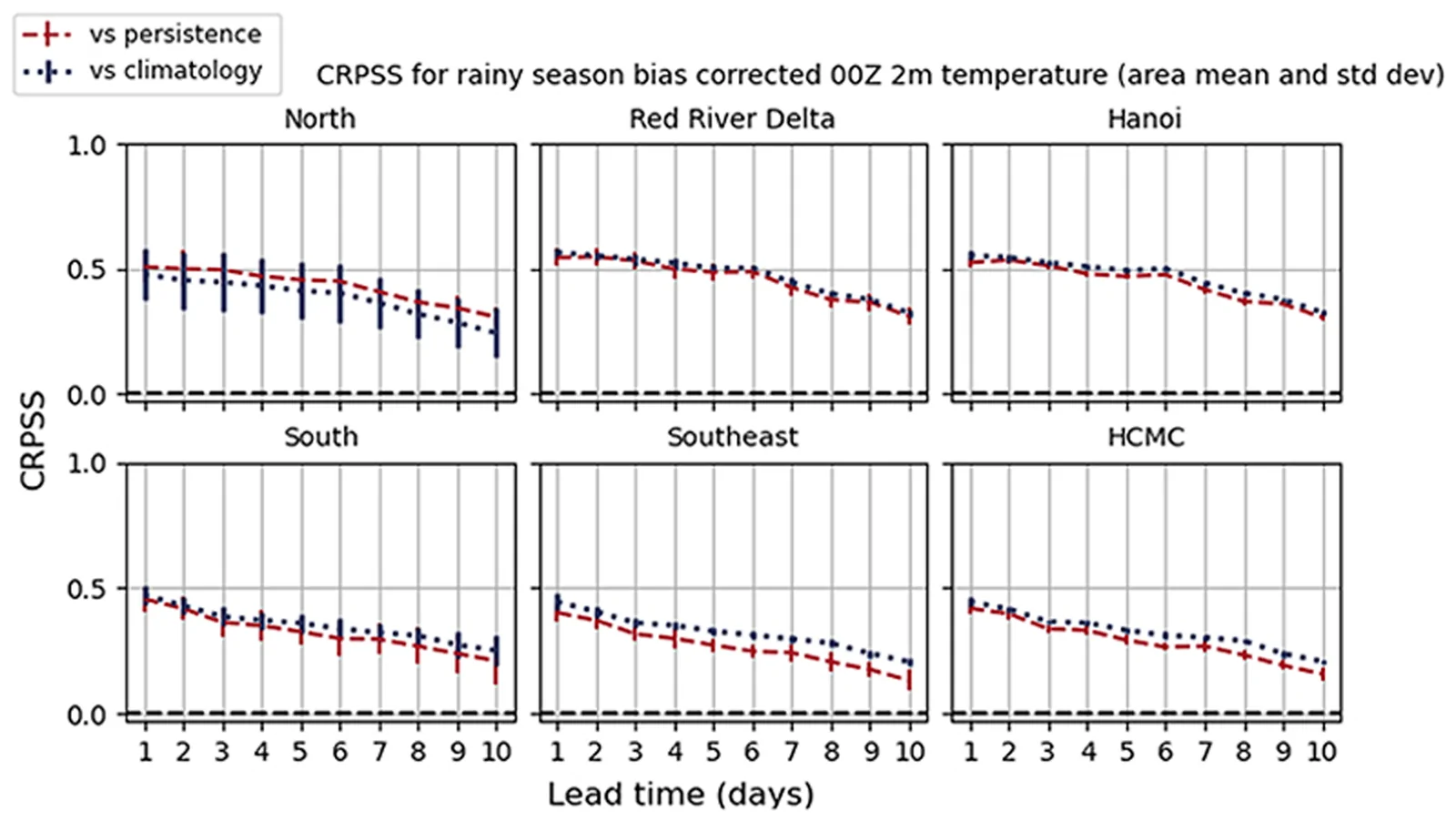
Rainy season (May–October) 00Z 2-m temperature CRPSS values (after bias correction) for each location and scale with lead times up to 10 days, showing both persistence (red) and climatology (blue) as reference forecasts. Skill is higher in the North than the South, but all locations are still skilful after 10 days for both persistence and climatology as reference forecasts, with scores remaining above around 0.2. Compared with Figures 11 and 12, there is less difference between results when climatology and persistence are used as references; mean persistence skill scores are slightly lower for all locations except the North macro-region, where the relatively large standard deviation for climatology indicates more variability in skill across the area (as shown in Figure S6).

00Z temperature CRPSS performance in Figure 13, using bias-corrected values, is skilful overall, with much less difference between persistence (red) and climatology (blue) as references than for precipitation. It is likely that the consistently high autocorrelation (Figure 5b) for both macro-regions makes persistence a more competitive reference for temperature. Values are generally in the range of 0.2–0.5 and larger in North Vietnam than South Vietnam, but all locations are still skilful after 10 days. As with precipitation, reducing spatial scale does not change mean skill considerably while reducing the standard deviation of CRPSS values. CRPSS against the climatology reference decays at a slower rate than for daily precipitation (Figure 11), and suggests that forecasts beyond 10 days may also be skilful.

Maps of 00Z temperature CRPSS values with a climatology reference (Figures S5 and S6) show there is spatially-dependent improvement when QM is used for bias correction. Before correction, scores in the North are worse to the west. Scores in the South are poor across the whole area, showing no evidence of skill compared with climatology except right by the coast. Following bias correction, the spatial uniformity of CRPSS values in North Vietnam increases and the decline of scores with lead time seems to be less marked on the whole. Improved performance in the South is more obvious, as CRPSS values become positive across the macro-region, including the area around HCMC. CRPSS is generally in the range of 0.1–0.5, with a gradual decline with lead time.

### 4.4 VnGP

**Figure 14:**
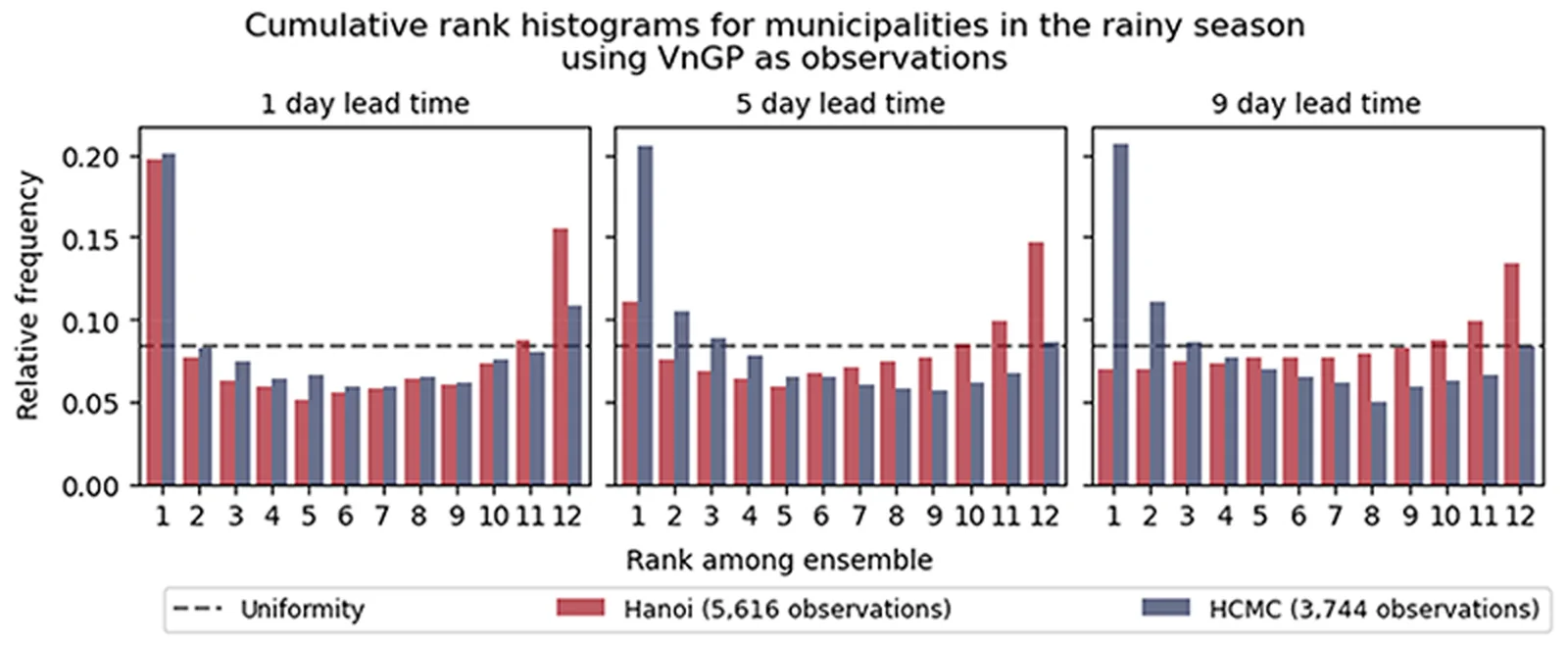
VnGP cumulative precipitation rank histograms for 1-, 5- and 9-day lead times over Hanoi (red) and HCMC (blue) in the rainy season (May–October). There is generally more overconfidence than when ERA5 is used as observational data (Figure 9). For Hanoi, overconfidence transitions to underforecasting as lead time increases, whereas HCMC shows a more consistent overpopulation of the first rank indicative of overforecasting. Note that when comparing with VnGP, an *x*-day lead time includes an additional 12 h (i.e. 12 Þ 24 × *x* hours in total) to match re-forecast and observation times, and that there are fewer observations as only every other re-forecast grid point has VnGP data.

Verified against independent local observations, forecasts again show significant and substantial skill against both persistence and climatology reference forecasts. Cumulative precipitation rank histograms using VnGP as observations rather than ERA5 (compare Figure 14 with Figure 9) are more overconfident, with a lack of spread to span the full range of observations: this might be because re-forecasts and observations are no longer generated from a similar underlying model. The 5- and 9-day histograms for Hanoi are more uniform than the 1-day histogram, which suggests that forecasts at short lead times are under-dispersive. The uniformity of forecasts at longer lead times reflects positively on their reliability and usefulness as inputs to the DART system. Rank histograms are similar for larger spatial scales (not shown).

**Figure 15:**
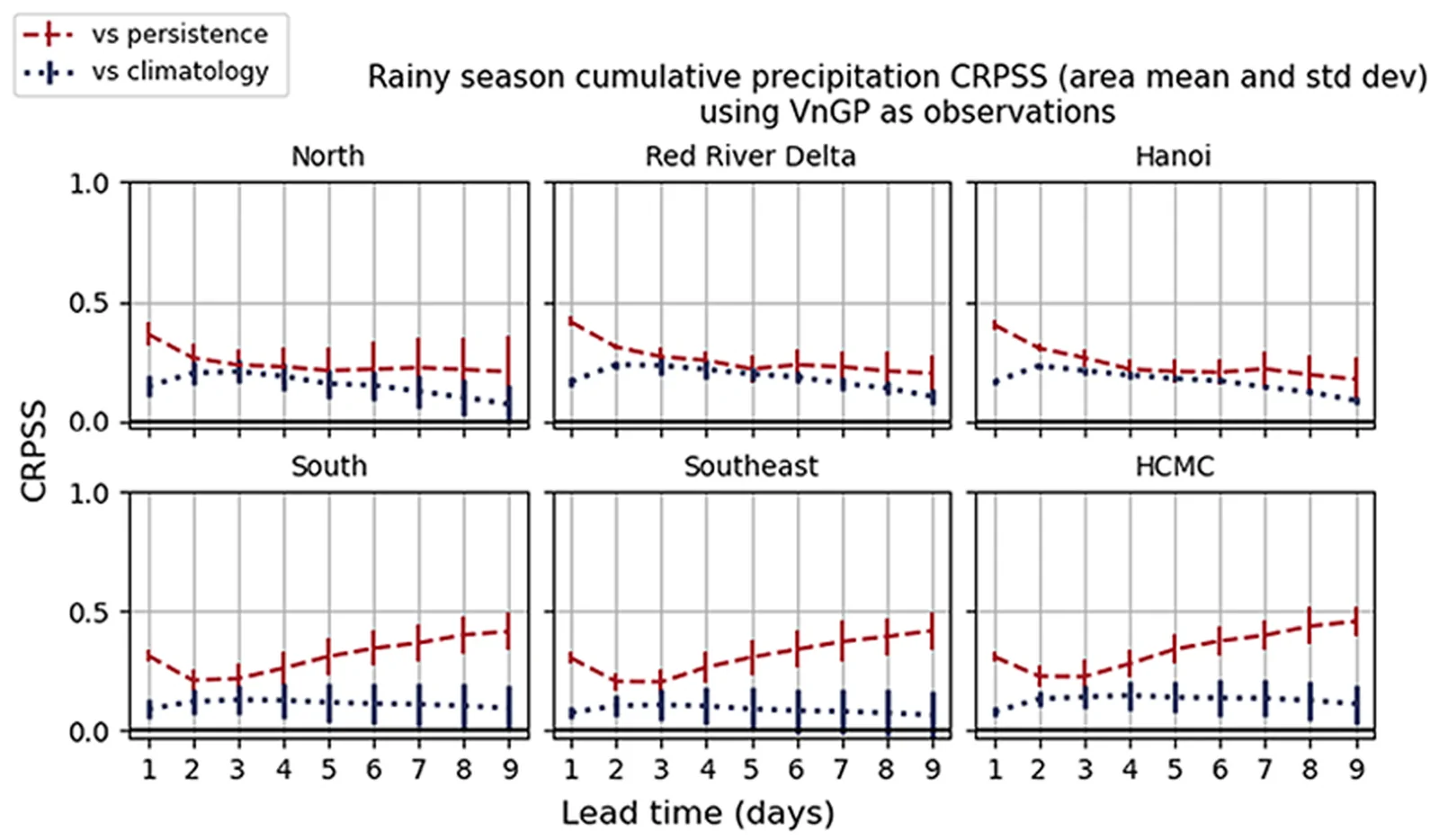
Rainy season (May–October) cumulative precipitation CRPSS values for each location and scale with lead times up to 10 days when VnGP is used as observational data rather than ERA5. Both persistence (red) and climatology (blue) are shown as reference forecasts. Compared with Figure 12, skill scores are reduced for both persistence and climatology at all lead times and locations, but remain positive to suggest that skill is retained at lead times beyond a week. Hence using VnGP as independent observations appears to corroborate the skill of the cumulative precipitation forecasts for the lead times considered. The same differences between North and South seen when using ERA5 for verification can also be observed, particularly the increase in skill compared with persistence in the South. Note that each lead time is 12 h longer than when comparing with ERA5 observations as a result of offsets between the datasets.

Cumulative precipitation CRPSS values using VnGP as observations are positive for all lead times considered when verifying against both persistence and climatology. Compared with ERA5 in Figure 12, the CRPSS values (vs. climatology) in Figure 15 are consistently lower, but with an even more gradual decline with lead time. At all locations, there is still skill after 10 days. Skill relative to persistence (red) shows the same difference between North and South as is seen using ERA5 as observations, with an increase in skill after the second day in the South and a very gradual decline in the North. As with climatology, CRPSS values are lower than when using ERA5, but skill is still apparent. Differences when using VnGP are to be expected, as the observations are derived from interpolation of rain gauge data rather than a similar underlying model; spatial offsets between the two datasets may also account for some differences.

A trend for scores to increase slightly after the first day can be seen for the cumulative precipitation CRPSS values (vs. climatology, in blue) using VnGP in Figure 15, perhaps relating to the under-dispersion seen in the rank histograms. Ensemble prediction systems are designed with the medium range in mind and can struggle to create spreads that adequately capture uncertainty in the first day or so (Arribas et al., 2005). Under-dispersion at 1-day lead times has been observed in other tropical and subtropical locations (Vogel et al., 2020). It could also be related to initialization shock. These shocks can arise, for example, from separate assimilation of ocean and atmosphere observations into the forecasting model, where imbalances in the initial state can lead to forecast errors (Mulholland et al., 2015).

## 5 Discussion and Conclusions

### 5.1 Summary and discussion of results

For all spatial scales considered, this study has demonstrated that ECMWF’s forecasts are significantly skilful in prediction of rainy season (May–October) precipitation and 2-m temperature over the 10-day forecast horizon, relative to both climatology and persistence. This is despite the different precipitation and temperature characteristics between North and South Vietnam and inter-annual variability of rainy season conditions. The skilful prediction of both precipitation and temperature by ECMWF’s forecasting system out to 10 days will, therefore, provide the DART system with reliable inputs.

Bias correction by QM was effective at removing a systematic cold bias in 2-m temperature values. Reforecast reliability was improved by this bias correction of temperature values, shown through the flattening of rank histograms and increased CRPSS, although with some spatial dependence in the size of the improvement.

ECMWF re-forecasts showed significant skill relative to persistence for lead times of 1–10 days. For precipitation, CRPSS using persistence as a reference was consistently higher, indicating that climatology was the harder reference forecast to beat; particularly in the South, with lower precipitation temporal autocorrelation, skill generally showed an increase with lead time as persistence ensembles became less skilful reference forecasts. In both locations, using cumulative rather than daily precipitation totals demonstrated notably less decline in area mean CRPSS scores calculated with climatology, and re-forecasts showed skill for lead times of up to 10 days. This is valuable to dengue modelling and the DART forecasting tool, as it suggests that cumulative precipitation re-forecasts made 1 or 2 weeks in advance are likely to be skilful inputs. CRPSS values for 00Z temperature were higher than for daily precipitation, and also showed skill for the whole 10-day period. The ability to incorporate skilful 10-day forecast information into the DART system (as opposed to current observational weather inputs) offers the potential for skilful disease predictions to be made 10 days further in advance. This is valuable lead time that could enable targeted interventions to be made, increase preparedness of hospitals and local authorities for impending outbreaks and inform the general public of upcoming risks.

CRPSS values indicated that forecasting over the specified smaller spatial scales around Hanoi and HCMC did not show any obvious reduction in reforecast skill for a given lead time; in fact, the standard deviation of CRPSS scores across the areas generally decreased with the size of the spatial scale. This might be because smaller-scale behaviour is dominated by large-scale monsoon dynamics that the model is able to closely replicate. However, this study did not require the model to replicate the exact physics of precipitation events below scales of around 28 km. It would be unlikely that localization of events to the urban scale would follow from effective forecasting of monsoon dynamics as a whole, given the need to parameterize convection, radiation and cloud microphysics.

While its spatial and temporal completeness, coupled with a global coverage, makes the reanalysis precipitation product a valuable initial benchmark for establishing skill, the consistent reduction in CRPSS values (compared with both climatology and persistence) when using VnGP in place of ERA5 indicates that ERA5 cannot be considered an authoritative proxy for rain gauge observations. Using VnGP as observed precipitation increased the overconfidence seen in rank histograms but did not suggest a lack of skill, as all CRPSS values were still positive at a 10-day lead time. The slight increase in VnGP CRPSS against climatology between 1- and 2-day lead times could suggest that the spread of ensembles at 1-day lead times is not sufficiently large, or be indicative of initialization shock.

Daily precipitation CRPSS scores (vs. climatology and using ERA5 as verification) exceeded previous evaluations of ECMWF outputs, where skill started at around 0.3 and fell below 0.1 after 7 days when extra-tropic skill was computed versus climatology for the whole year, August 2021–July 2022, over all available stations (Haiden et al., 2022). This could be indicative of the predictability monsoon circulation brings to rainy season in Vietnam, but it may also be influenced by underlying model similarity between the re-forecasts and ERA5.

Figure 23 in Haiden et al. (2022) indicates a daytime cold bias in 2-m temperature forecasts against European SYNOP data, with similar magnitude to the biases over Vietnam shown here, notwithstanding the different processes in the tropics and extra-tropics that could account for them. Cold biases have also been identified towards the southeastern part of the Qinghai-Tibet Plateau (Wei et al., 2021), around 5 degrees north of Vietnam’s border with China. The forecast lead time for which 850 hPa temperature CRPSS (vs. climatology) reaches 0.25 in the Northern Hemisphere extra-tropics is around 9 days (Haiden et al., 2022). Direct comparison to this result is not possible, as this study uses 2-m temperature, but this is similar to the results seen for 00Z temperature for both North and South Vietnam.

Reanalysis and observation data, like NWP forecasts, have uncertainties associated with them. An important limitation in this study and its results is the assumption that uncertainties in the ERA5 and VnGP datasets are negligible, when in reality they are limited by the need to constrain spatially continuous data with discrete observations. Stationary climatology has also been assumed when using climatology as a reference forecast and in the temperature bias correction procedure when constructing the ERA5 CDF. There was no obvious trend over the two decades’ worth of data used to suggest a change in climatology.

### 5.2 Further work

The skill demonstrated in this study is already being leveraged by the interdisciplinary team in the DART project to develop a prototype dengue early warning system. Bias-corrected temperature and accumulated precipitation outputs from ECMWF re-forecasts are being used as inputs during the development of a new epidemiological model. Further work is ongoing to evaluate the skill of humidity in the re-forecasts, another important variable in vector-borne disease transmission (Brown et al., 2023). Considering the skill at times of the day most influential to mosquito biting behaviours, such as around dawn and dusk or in relation to artificial light in urban areas (Rund et al., 2020), could likewise provide additional value.

Furthermore, the results from this study showing skill up to 10-day lead times suggest that sub-seasonal forecasts of 2-m temperature and accumulated precipitation may provide skilful inputs into the DART system. Dependent on the factors that dominate the predictions made by the prototype dengue early warning system, further work could investigate sub-seasonal skill, exploring whether forecasts made around 2–4 weeks ahead demonstrate skill compared with climatology (which is likely a harder reference to beat at sub-seasonal lead times). This would be profitable if meteorological inputs were found to have a prevailing effect on dengue forecasts. In that case, it would be worth investigating the sources of monsoon predictability (e.g. ENSO, MJO, soil moisture) that might lead to skill at these lead times.

Changing the bounding boxes used to define each spatial scale, or masking the sea in the evaluation of macro-region skill, could test the sensitivity of skill to spatial scale, and validate the generalizability of this study’s findings. The positive CRPSS values presented in this study indicate a high baseline of skill on which localized, urban forecasts for dengue modelling might build with statistical and/or dynamical downscaling techniques. This is particularly the case because results at municipal and regional scales were no less skilful than North and South Vietnam overall, and indeed generally showed a smaller range of CRPSS values with smaller standard deviations. The development of appropriate downscaling techniques is another focus of the DART project.

## Supplementary Material

Supplementary Information

## Figures and Tables

**Table 1:** Summary of observation datasets used in this study. ERA5 high-resolution reanalysis has a single member.

Observation dataset	Resolution	Spatial extent	Date range	Units	Source
ERA5 daily total precipitation	0.25° (~28 km)	5–25° N and 100–110° E	01/01/00 to 31/10/22	mm (from m)	ECMWF
ERA5 00Z 2 m temperature	0.25° (~28 km)	5–25° N and 100–110° E	01/01/01 to 31/12/20	°C (from K)	ECMWF
Vietnam Gridded Precipitation (VnGP)	0.1° (~11 km)	8–24° N and 100–112° E	01/01/00 to 31/12/18	mm	University of Science and Technology of Hanoi

**Table 2:** Summary of the re-forecast datasets used in this study. Resolution is the grid the data are made available on, not the native resolution. The IFS ENS re-forecasts are retrospective forecasts provided for skill assessment and calibration; more information is available table 1b in ECMWF (2023).

Forecast dataset	Resolution	Extent	Dates	Units	Source	Forecast range	Initialization	Members
IFS ENS re-forecast 00Z 2-m temperature	0.25° (~28 km)	5–25° N and 100–110° E	04/01/01 to 30/12/20	°C (from K)	ECMWF	0 – 240 h	Twice weekly	1 control +11 perturbed
IFS ENS re-forecast daily total precipitation	0.25° (~28 km)	5–25° N and 100–110° E	04/01/01 to 30/12/20	mm (from m)	ECMWF	0 – 240 h	Twice weekly	1 control +11 perturbed

**Table 3:** Summary of the orography data used in this study. These data are on the same horizontal grid and at the same resolution as the re-forecasts and reanalysis.

Orography dataset	Resolution	Spatial extent	Units	Source
Geopotential height	0.25° (~28 km)	5–25° N and 100–110° E	m	ECMWF

## Data Availability

The data that support the findings of this study are available from the corresponding author upon reasonable request.
