# Supplementary material for: Skilful probabilistic medium-range precipitation and temperature forecasts over Vietnam for the development of a future dengue early warning system
Source: Meteorol Appl. Author manuscript; Available in PMC 2026 Oct 30. (PMC7619663; doi:10.1002/met.2222)
Supplement: Supplementary file 1 [file EMS218497-supplement-Supplementary_Information.docx]

| **Spatial Scale** | **Name** | **Latitude (°N)** | **Longitude (°E)** | **Approx. spatial extent (horizontal x vertical, km^2^)** |
| --- | --- | --- | --- | --- |
| Macro-region | North Vietnam | 20.25-23.5 | 102-108 | 672 x 364 |
|  | South Vietnam | 8.5-12 | 104.25-107.5 | 364 x 392 |
| Region | Red River Delta | 20.25-21.5 | 105.5-106.75 | 140 x 140 |
|  | Southeast | 10.5-12 | 106-107.5 | 168 x 168 |
| Municipality | Hanoi | 20.5-21.5 | 105.5-106 | 56 x 112 |
|  | Ho Chi Minh City | 10.5-11.25 | 106.25-107 | 84 x 84 |

Table S1

Latitudes and longitudes defining the bounding boxes used for each location considered.

Figure S1

Violin plots showing the distribution of 00Z temperature values for each month in North Vietnam. The ensemble is shown in red and the reanalysis (ERA5) in blue. The distributions are typically similar in shape but there is a systematic cold bias where ensemble values are consistently lower by around 0.5-1°C. Bias is similar in South Vietnam, which is not shown.

Figure S2

Violin plots showing the distribution of 00Z temperature values for each month in North Vietnam after bias correction with quantile mapping. Compare with Figure S1 to see the achieved reduction in cold bias. Similar results are achieved for South Vietnam.

Figure S3

Rank histograms for rainy season (May-October) 00Z 2m temperature at 1, 4, 7 and 10-day lead times before bias correction over Hanoi (red) and HCMC (blue). The re-forecasts show strong underforecasting tendencies arising from the systematic cold bias, with a particularly blatant favouring of the 12^th^ rank for HCMC.

Figure S4

Maps of the mean CRPSS (vs climatology) values for cumulative precipitation during the rainy season (May-October) for 1, 4, 7 and 10-day lead times. a) North Vietnam. Performance declines with increasing lead time, with a noticeable drop to well below 0.4 across the whole macro-region after 10 days. b) South Vietnam. The reduction in skill is less stark than over North Vietnam, as 1-day scores are typically a lower starting point, with the same general decreasing trend; scores west of HCMC appear to be generally poorer.

Figure S5

Maps of the mean CRPSS (vs climatology) values for uncorrected (raw) 00Z 2m temperature during the rainy season (May-October) for 1, 4, 7 and 10-day lead times. a) North Vietnam. Performance is better in the flatter area around Hanoi and poorer in the mountainous northwest. b) South Vietnam. Performance is uniformly poor, showing almost no evidence of skill across the whole macro-region except for along the coastlines with the South China Sea and Gulf of Thailand.

Figure S6

Maps of the mean CRPSS (vs climatology) values for bias corrected 00Z 2m temperature during the rainy season (May-October) for 1, 4, 7 and 10-day lead times. a) North Vietnam. Comparison with Figure S5a shows that bias correction has increased the spatial uniformity of skill scores, with particular improvement for grid points in the more mountainous west. There also appears to have been a reduction in the decline of skill with lead time. b) South Vietnam. Comparison with Figure S5b indicates that bias correction successfully renders the re-forecasts skilful relative to climatology, although values are overall lower than for North Vietnam. A decrease in skill with lead time also becomes apparent.
